# Lipophilic signals lead to organ‐specific gene expression changes in *Arabidopsis* seedlings

**DOI:** 10.1002/pld3.242

**Published:** 2020-07-15

**Authors:** Ashley E. Cannon, Chengshi Yan, David J. Burks, Xiaolan Rao, Rajeev K. Azad, Kent D. Chapman

**Affiliations:** ^1^ BioDiscovery Institute and Department of Biological Sciences University of North Texas Denton TX USA; ^2^ Department of Mathematics University of North Texas Denton TX USA

**Keywords:** *Arabidopsis* thaliana, lipids, *N*‐acylethanolamines, RNA‐seq, secondary dormancy, seedling development

## Abstract

In plants, *N*‐acylethanolamines (NAEs) are most abundant in desiccated seeds and their levels decline during germination and early seedling establishment. However, endogenous NAE levels rise in seedlings when ABA or environmental stress is applied, and this results in an inhibition of further seedling development. When the most abundant, polyunsaturated NAEs of linoleic acid (18:2) and linolenic acid (18:3) were exogenously applied, seedling development was affected in an organ‐specific manner. NAE 18:2 primarily affected primary root elongation and NAE 18:3 primarily affected cotyledon greening and expansion and overall seedling growth. The molecular components and signaling mechanisms involved in this pathway are not well understood. In addition, the bifurcating nature of this pathway provides a unique system in which to study the spatial aspects and interaction of these lipid‐specific and organ‐targeted signaling pathways. Using whole transcriptome sequencing (RNA‐seq) and differential expression analysis, we identified early (1–3 hr) transcriptional changes induced by the exogenous treatment of NAE 18:2 and NAE 18:3 in cotyledons, roots, and seedlings. These two treatments led to a significant enrichment in ABA‐response and chitin‐response genes in organs where the treatments led to changes in development. In *Arabidopsis* seedlings, NAE 18:2 treatment led to the repression of genes involved in cell wall biogenesis and organization in roots and seedlings. In addition, cotyledons, roots, and seedlings treated with NAE 18:3 also showed a decrease in transcripts that encode proteins involved in growth processes. NAE 18:3 also led to changes in the abundance of transcripts involved in the modulation of chlorophyll biosynthesis and catabolism in cotyledons. Overall, NAE 18:2 and NAE 18:3 treatment led to lipid‐type and organ‐specific gene expression changes that include overlapping and non‐overlapping gene sets. These data will provide future, rich opportunities to examine the genetic pathways involved in transducing early signals into downstream physiological changes in seedling growth.

## 1 INTRODUCTION

*N*‐Acylethanolamines (NAEs) are fatty acid derivatives with ethanolamine conjugated via an amide linkage, and these lipids have been identified in plants, animals, and some microorganisms (Blancaflor et al., 2014). In animal systems, the most studied NAE is *N*‐arachidonylethanolamine or anandamide (Zou & Kumar, 2018). NAEs are referred to based on the length of the acyl chain and the number of double bonds. For example, anandamide is composed of 20 carbons and four double bonds in the acyl portion of the molecule, leading to the designation, NAE 20:4. NAE 20:4 is an endogenous ligand for the two primary cannabinoid (CB) receptors, CB1 (Devane et al., 1992) and CB2 (Munro, Thomas, & Abu‐Shaar, 1993), and consequently its metabolic formation and action define the endocannabinoid signaling pathway (Iannotti, Di Marzo, & Petrosino, 2016).

Although the role of NAEs and the endocannabinoid signaling pathway in animal systems is becoming well understood, our knowledge about these lipophilic signals and the corresponding transduction pathways in plants is limited. In plant systems, the length of the acyl chain and the degree of saturation of the NAE is dependent on the developmental stage, environmental condition(s), and the tissue(s) assessed (Blancaflor et al., 2014; Chapman, 2004; Kilaru et al., 2007). NAEs have been identified in several plant species and the highest concentrations occur in desiccated seeds (Chapman, 2004; Venables, Waggoner, & Chapman, 2005). The levels of NAEs decline during germination and seedling establishment coincidentally with abscisic acid (ABA) (Teaster et al., 2007), and studies have indicated an interaction between NAE and ABA signaling (Cotter, Teaster, Blancaflor, & Chapman, 2011; Keereetaweep, Blancaflor, Hornung, Feussner, & Chapman, 2015).

NAEs can be hydrolyzed by fatty‐acid amide hydrolase (FAAH) (Aziz, Wang, Tripathi, Bankaitis, & Chapman, 2019; Kim, Faure, & Chapman, 2013; Shrestha, Noordermeer, van der Stelt, Veldink, & Chapman, 2002; Wang et al., 2006) or oxidized by lipoxygenase (LOX) enzymes (Kilaru et al., 2011) and these two metabolic processes result in the production of several conjugated or free‐fatty acid and oxylipin species that may be involved in the modulation of plant growth and development (Blancaflor et al., 2014; Cotter et al., 2011; Hofmann, 2013; Keereetaweep, Blancaflor, Hornung, Feussner, & Chapman, 2013; Keereetaweep et al., 2015; Kilaru et al., 2007; Teaster et al., 2007). Previous studies have shown that exogenous NAE treatment leads to an inhibition in seedling growth, changes in cytoskeleton and endomembrane organization, and chlorophyll degradation (Blancaflor et al., 2014; Keereetaweep et al., 2013, 2015; Teaster et al., 2007). More specifically, exogenous treatment with NAE 18:2 or NAE 18:3, the two most abundant polyunsaturated NAEs in *Arabidopsis*, leads to an inhibition in primary root growth or an overall decrease in seedling growth and cotyledon de‐greening, respectively (Keereetaweep et al., 2013, 2015).

*Arabidopsis* seedlings are sensitive to the effects of these two NAEs during a specific window of seedling development when seedlings are also ABA sensitive (Keereetaweep et al., 2013, 2015; Lopez‐Molina, Mongrand, & Chua, 2001). During this window of sensitivity, an increase in NAE 18:2, NAE 18:3, or ABA levels leads to the induction of an ABA‐dependent, post‐germinative process referred to by Lopez‐Molina *et al*. as secondary dormancy (Keereetaweep et al., 2013, 2015; Lopez‐Molina et al., 2001). During secondary dormancy, growth is inhibited, metabolism is slowed, and cellular components are recycled (Lopez‐Molina et al., 2001), until conditions are more favorable for development and growth resumes. The NAE 18:2‐mediated induction of this process was shown to require an intact ABA‐signaling pathway (Keereetaweep et al., 2015). Collectively, these studies describe in part a lipid‐mediated pathway in *Arabidopsis* that interacts with the ABA signaling pathway to modulate seedling establishment through the post‐germinative survival mechanism of secondary dormancy.

In addition to playing a role in germination and seedling establishment, NAEs are also involved in plant–microbe interactions (Aziz & Chapman, 2020; Blancaflor et al., 2014; Kang et al., 2008; Kim, Chapman, & Blancaflor, 2010; Tripathy, Venables, & Chapman, 1999). NAEs accumulated in the growth media of tobacco cell suspensions as a result of treatment with xylanase, a fungal elicitor (Tripathy et al., 1999). In addition, the ectopic overexpression of the NAE degrading enzyme, FAAH, made *Arabidopsis* plants more susceptible to bacterial pathogens (Kang et al., 2008). In these FAAH overexpressing plants, enhanced susceptibility to pathogens coincided with a decrease in expression of defense‐related transcripts (Kang et al., 2008). In addition to modulating the defense response, the NAE metabolism machinery is also involved in the plant response to bacterial or fungal NAEs or NAE‐like compounds (Aziz & Chapman, 2020; Palmer, Senechal, Mukherjee, Ané, & Blackwell, 2014; Zhang et al., 2019). For example, a fungal‐derived NAE, NAE 12:0, has also been shown to interact with components of the NAE metabolic pathways and initiate physiological changes in plants (Zhang et al., 2019). Inoculation of cotton seedlings with *Verticillium dahliae* led to an increase in transcript levels of four FAAH enzymes, a decrease in seedling growth, and changes in the cytoskeleton (Zhang et al., 2019). *V. dahliae* produces many secondary metabolites, including NAE 12:0, that cause defoliation in cotton by altering pathogen sensitivity or disrupting hormone signaling (Zhang et al., 2019). Consequently, a picture is emerging where pathogen‐derived NAEs or NAE‐like molecules added to plants elicit changes in NAE metabolism and cause physiological changes that are similar to the developmental changes induced by the exogenous application of NAEs.

In general, results to date suggest a role for NAE metabolism in mediating plant growth and stress responses. However, the molecular components and signaling mechanisms involved in these processes have not been fully defined. There is a clear interaction between NAE 18:2 and the NAE 18:2‐derived oxylipin, 9‐hydroxy octadecadienoyl ethanolamide (9NAE‐HOD), with ABA signaling (Keereetaweep et al., 2015). However, involvement of ABA is less clear in NAE 18:3‐dependent inhibition of seedling growth which includes the reduction in cotyledon expansion and depletion of chloroplasts. Consequently, there must be additional pathways involved in mediating the responses of cotyledons to NAE 18:3. In addition, the actions of NAE 18:2 are restricted to roots of seedlings; cotyledons were not visibly affected (Keereetaweep et al., 2015). In contrast, NAE 18:3 affects both roots and cotyledons of seedlings (Keereetaweep et al., 2013). Based on the bifurcating nature of this pathway, it appears that there may be lipid‐type and organ‐specific molecular changes that precede the phenotypic changes induced by NAE18:2 and NAE 18:3 in early seedling establishment. Considering that both of these NAEs are derived from the same precursor pool in desiccated seeds (Chapman, 2004; Teaster et al., 2007), it is interesting to consider the mechanism(s) that might confer the selective actions in different organs.

In order to gain insights into how NAE 18:2 and NAE 18:3 modulate organ‐specific molecular changes, we sequenced transcripts from dissected cotyledons or roots after a 1–3 hr treatment with exogenous NAE 18:2 or NAE 18:3, and compared these organ‐specific changes in gene expression to those of seedlings. Using these RNA‐seq data, we identified lipid signal‐type and organ‐specific gene expression changes that likely initiate or contribute to the downstream phenotypic changes that follow the exogenous treatment with these two seedling growth mediators.

## 2 METHODS

### 2.1 *Arabidopsis* seedling growth, treatment, and plant organ collection

*Arabidopsis* Col‐0 seeds (Lehle Seeds) were surface sterilized by treatment with 70% ethanol for 3 min followed by 20% bleach (5.25% sodium hypochlorite, Clorox) with 0.01% Triton X‐100 (Sigma‐Aldrich) for 5 min and then rinsed five times with sterile ddH2O. After the final rinse, *Arabidopsis* seeds were maintained in 1 ml of sterile ddH2O, wrapped in two layers of aluminum foil, and placed in the dark at 4°C for 3 days. After 3 days, the seeds were sown in 50 ml of 1/2 Murashige and Skoog (Sigma‐Aldrich) media, pH 5.7 with 1% sucrose (Fisher Scientific) in a 250 ml sterile Erlenmeyer flask. The seeds were grown at 21°C, 100 µmoles/m^2^s, on a shaker (Bellco Biotechnologies) set at speed level 3.

After 3 days, the seedlings were separated into groups of 50 using sterile toothpicks and moved to 25 ml of 1/2 Murashige and Skoog in a petri dish (100 × 25 mm, Fisher Scientific). The seedlings were treated with 0.2% DMSO (Sigma‐Aldrich), 40 µM NAE 18:2 (Cayman Chemical), or 70 µM NAE 18:3 (Cayman Chemical) for 1–3 hr. After 1–3 hr, whole seedlings, roots, and cotyledons were collected from each treatment group, flash frozen, and stored at −80°C.

Seedlings used for root growth and chlorophyll content assays were imaged after 3 days. Seedlings were imaged using a Nikon D5100 digital camera and primary root length was measured using ImageJ software (Keereetaweep et al., 2015; Schneider, Rasband, & Eliceiri, 2012). Chlorophyll extraction and quantification were done using the methods described in Keereetaweep et al. (2013). Primary root lengths and total chlorophyll content of treatment groups were compared using a one‐way ANOVA and a post‐hoc Tukey Honestly Significant Difference (HSD) test in Excel.

### 2.2 RNA Isolation, cDNA library preparation, Illumina sequencing

Three biological replicates of each *Arabidopsis* organ (cotyledons or roots) or seedling sample from each treatment condition (0.2% DMSO, 40 µM NAE 18:2 or 70 µM NAE 18:3) were ground to a fine powder and 500 µl of Hot Borate Buffer (200mM sodium tetraborate decahydrate—Sigma‐Aldrich, 30 mM EGTA—Sigma‐Aldrich, 1% SDS—Fisher, 1% deoxycholic acid—Sigma‐Aldrich, 2% polyvinylpyrrolidone (PVP) 40K—Sigma‐Aldrich, 0.5% IGEPAL CA‐630—Sigma‐Aldrich, and 10 mM DTT—Sigma‐Aldrich, 80°C) and 17.5 µl of 20 mg/ml Proteinase K (ThermoFisher) were added (Wu, Llewellyn, & Dennis, 2002). After mixing, the sample and buffer solution were added to a lilac shredder column from a RNeasy Plant Mini Kit (Qiagen). The manufacturer's protocol from the RNeasy Plant Mini Kit was followed for the remainder of the extraction. RNA quantity and quality were determined using the Quibit Fluoremeter (Invitrogen) and the 2100 Bioanalyzer (Agilent). Poly(A) enrichment and library preparation were performed using the TruSeq Stranded mRNA prep kit (Illumina). Libraries were quantified by fluorometry, immobilized, and processed onto a flow cell, followed by 2 × 75 bp sequencing by synthesis on a Next‐Seq 500 system (Illumina) with two mid‐output cassettes (Illumina). Library construction and RNA sequencing were performed by the UNT Genomics Center.

The quality of the RNA‐seq data was initially analyzed using FastQC. After initial analysis, any remaining adapters were trimmed using Trimmomatic (0.36 (Bolger, Lohse, & Usadel, 2014). Read quality was reassessed using FastQC and trimmed reads were mapped to the *Arabidopsis* genome (TAIR10) using STAR (Version 2.6) (Dobin et al., 2013) with the default settings. DESeq2 (Version 1.22.1) (Love, Huber, & Anders, 2014), with the default settings, was used for differential expression analysis and sample groups were compared by organ. Principal Component Analysis (PCA) was conducted in order to identify any potential outliers among the samples using pcaExplorer (Marini & Binder, 2016) (Additional File 12). The threshold for differentially expressed genes was set at False Discovery Rate (FDR; adjusted *p*‐value or q‐value) < 0.01 and a fold change > 2. The RNA‐seq data used in this study were deposited in the National Center for Biotechnology and can be accessed using accession number GSE134271.

### 2.3 Bioinformatic analyses of differentially expressed genes

GO enrichment analyses were performed using g:Profiler (Reimand et al., 2016), a publicly available tool for the functional interpretation of gene lists, and the GOsummaries (Kolde & Vilo, 2015) package was used to generate word cloud summaries in R (R Core Team, 2018). Venn diagrams were generated using Gene List Venn Diagram (http://genevenn.sourceforge.net/) or the Bioinformatics and Evolutionary Genomics Venn Diagram tool (http://bioinformatics.psb.ugent.be/webtools/Venn/). The AtGenExpress hormone treatment DataSet (Goda et al., 2008) was located on the NCBI GEO database (GEO accession GSE39384). The 1 hr control and 1 hr hormone treatment samples were compared using the NCBI GEO2R tool (Barrett et al., 2013) in order to identify Differentially Expressed Genes (DEGs). The hormone‐ (Goda et al., 2008) and chitin‐induced (Stringlis et al., 2018) differentially expressed genes were compared to NAE 18:2 and NAE 18:3‐induced genes in cotyledons, roots, and seedlings using the GeneOverlap program (Shen & Sinai, 2019) in R (R Core Team, 2018). Heat maps were generated using conditional formatting in Microsoft Excel or gplots v3.0.1.1 (Warnes et al., 2019) in R (R Core Team, 2018).

### 2.4 Validation of RNA‐seq data by quantitative real‐time PCR (RT‐qPCR)

To validate RNA‐seq data, the transcript levels of five genes were analyzed using real‐time quantitative PCR (RT‐qPCR) (Additional File 13). New RNA samples from three biological replicates of *Arabidopsis* seedlings from each treatment condition (0.2% DMSO, 40 µM NAE 18:2, or 70 µM NAE 18:3) were used for verification of transcript levels. The Applied Biosystems High Capacity cDNA Reverse Transcription Kit (Thermo Fisher) was used to synthesize cDNA using 1,000 ng of total RNA. The cDNA was diluted 1:10 ([10 ng/µL]) and used as a template in a 20 µl qPCR reaction using the Applied Biosystems PowerUp™ SYBR™ Green Master Mix (Fisher Scientific) following the manufacturer's protocol. The primer sequences used are shown in Additional File 13b. The two reference genes Glyceraldehyde‐3‐Phosphate Dehydrogenase C2 (GAPC2; AT1G13440) and Polyubiquitin 10 (UBQ10; AT4G05320) were chosen based on the work of Czechowski, Stitt, Altmann, Udvardi, and Scheible (2005) and the low level of variation between samples. The genes tested included Lipoxygenase 4 (LOX4; AT1G72520), Cytokinin Response GATA Factor 1 (CGA1; AT4G26150), LysM Receptor‐Like Kinase 5 (LYK5; AT2G33580), Xyloglucan Endotransglucosylase/hydrolase 13 (XTH13; AT5G57540), and the NAC Transcription Factor 072 (NAC072; AT4G27410). The transcript abundance of each gene was quantified using RT‐qPCR in an Applied Biosystems QuantStudio 3 Real‐Time PCR System using the Comparative Ct Program (ΔΔCt) (Thermo Fisher). The fold change of each gene was calculated in relation to the expression of the gene in the untreated samples using the 2^−ΔΔCT^ method (Livak & Schmittgen, 2001) (Additional File 13).

## 3 RESULTS

### 3.1 Comparisons of NAE 18:2 and NAE 18:3 treatment effects

**FIGURE 1:**
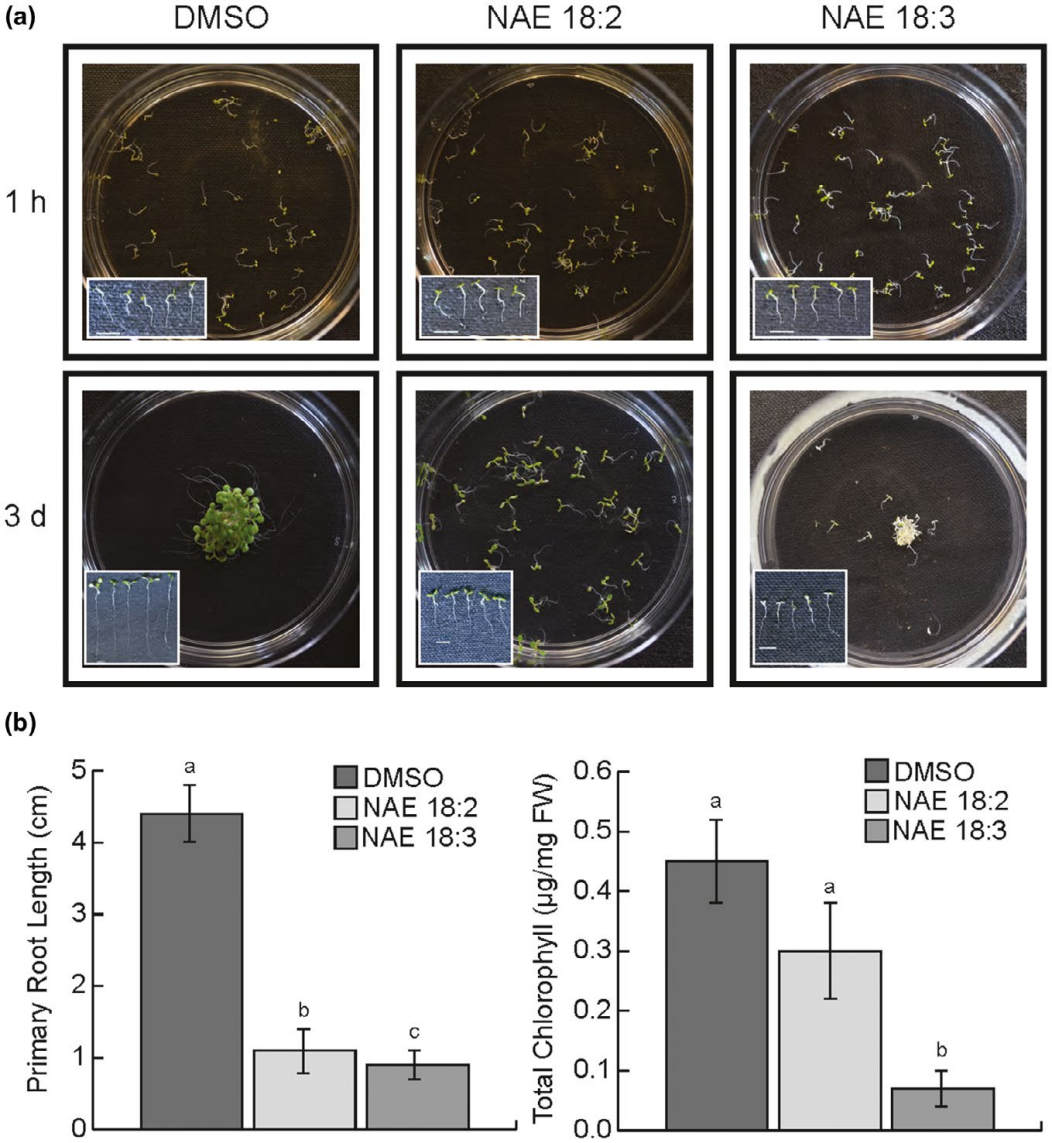
NAE 18:2 and NAE 18:3 treatment leads to phenotypic changes in 3‐day‐old *Arabidopsis* seedlings (a) *Arabidopsis* seedlings were treated with 0.2% DMSO, 40 µM NAE 18:2, or 70 µM NAE 18:3. After 1–3 hr of treatment, when the seedlings were collected for RNA‐seq, there were no visible differences. After 3 days of treatment, NAE 18:2 significantly decreased primary root length and NAE 18:3 significantly decreased primary root length and chlorophyll content. Scale bar = 0.5 cm in all images. (b) Primary root length (*n* = 20 or 30) and total chlorophyll content (*n* = 5–10) after 3 days of 0.2% DMSO, 40 µM NAE 18:2, or 70 µM NAE 18:3 treatment. A one‐way ANOVA showed that there was a significant decrease in primary root growth (*F* = 27.9, *p* = 9.0 × 10^−4^) and chlorophyll content (*F* = 1,091.7, *p* = 1.1 × 10^−16^) as a result of the NAE treatments. Post‐hoc analysis using the Tukey‐Kramer test showed that exogenous treatment with NAE 18:2 and NAE 18:3 caused a statistically significant decrease in primary root length and treatment with NAE 18:3 caused a statistically significant decrease in chlorophyll content

NAE 18:2 and NAE 18:3 treatment led to lipid‐type and organ‐specific effects when applied to 3‐ to 4‐day‐old *Arabidopsis* seedlings (Figure 1 and reviewed in (Blancaflor et al., 2014)). Treatment with 40 µM NAE 18:2 led to an inhibition in primary root elongation after 3 days, while treatment with 70 µM NAE 18:3 led to an inhibition in overall seedling growth and a decrease in the chlorophyll content of cotyledons (Figure 1a,b). Two different NAE concentrations were used because the amount of NAE 18:3 required to induce cotyledon de‐greening is higher than the amount of NAE 18:2 or NAE 18:3 required to inhibit primary root growth (Keereetaweep et al., 2013, 2015). Additionally, although the seedlings tend to clump together after 3 days of NAE treatment (Figure 1a), the NAE‐induced phenotypes are present in all treated seedlings and there is no evidence of this growth behavior affecting nutrient availability. These longer‐term changes in seedling growth are likely to be initiated by transcriptional changes much earlier, when there are no perceptible physiological differences (e.g., 1–3 hr; Figure 1a). Therefore, to analyze the effects of these treatments on transcript abundance and the role of NAE type and organ specificity in these changes, *Arabidopsis* seedlings were treated with NAE 18:2 or NAE 18:3 for 1–3 hr and whole seedlings, or dissected cotyledons and roots were collected for Illumina‐based, RNA sequencing analyses.

**FIGURE 2:**
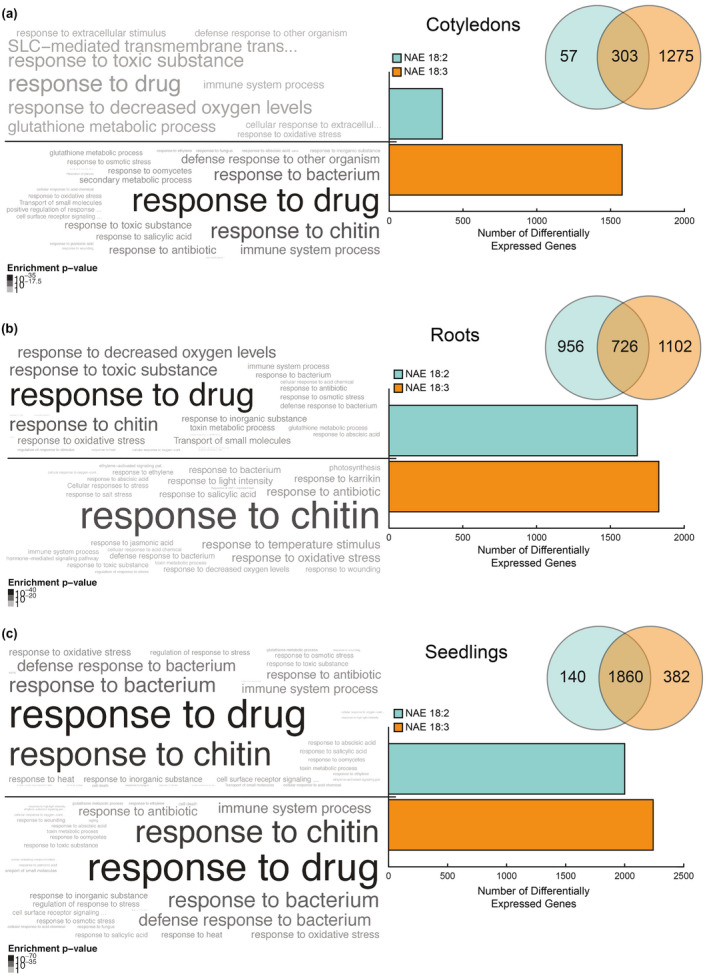
Genes induced by exogenous NAE 18:2 and/or NAE 18:3 treatment in cotyledons, roots, and seedlings. (a–c) Venn diagrams, bar graphs, and significantly enriched GO terms describing the genes induced by NAE 18:2 and/or NAE 18:3 in cotyledons (a), roots (b), and seedlings (c). Beside each bar, significantly enriched GO terms are displayed in word clouds above a p‐value color scale. The size and color of the term corresponds to the relative level of enrichment and the enrichment p‐value, respectively

**FIGURE 3:**
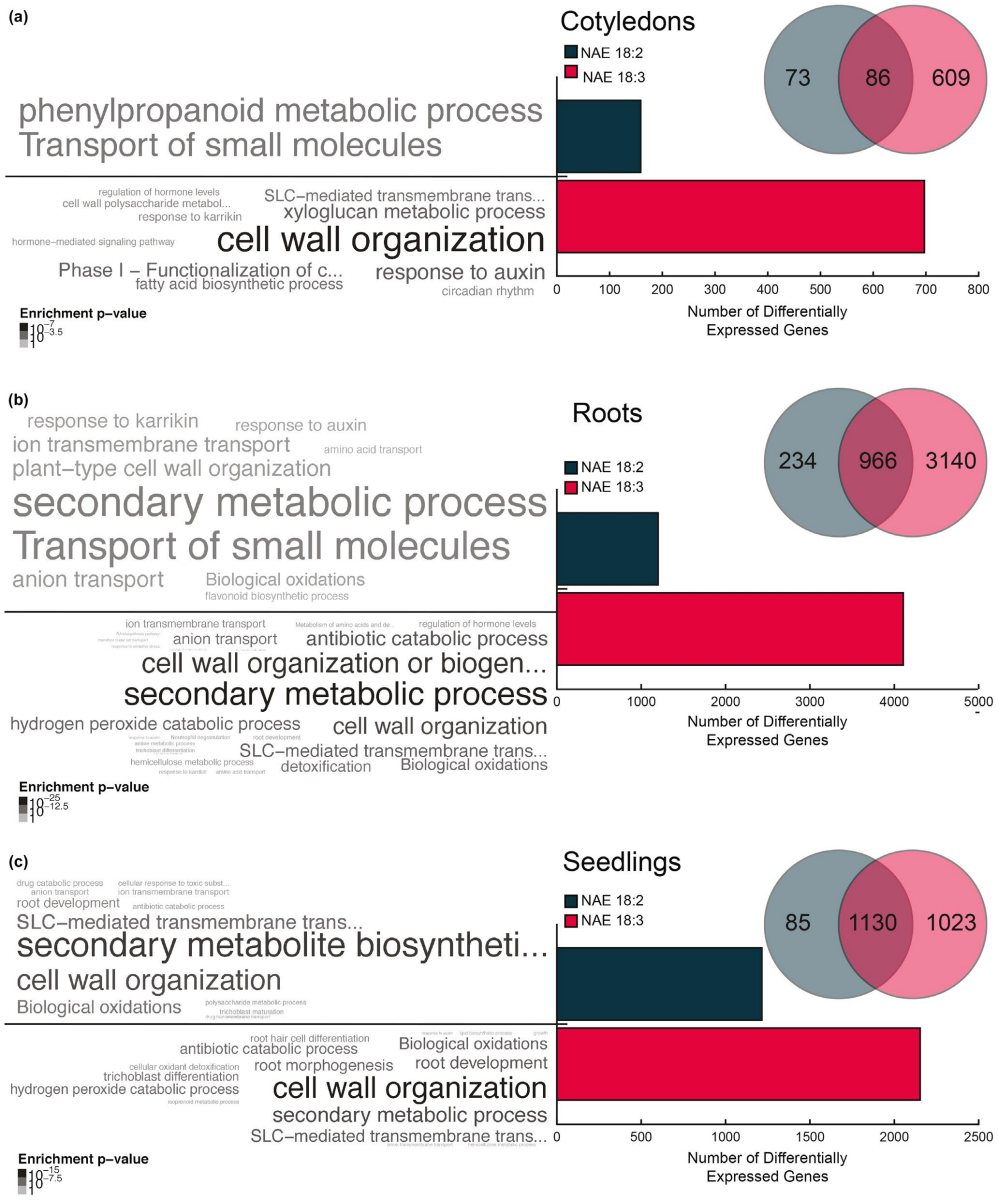
Genes repressed by exogenous NAE 18:2 and/or NAE 18:3 treatment in cotyledons, roots, and seedlings. (a–c) Venn diagrams, bar graphs, and significantly enriched GO terms describing the genes repressed by NAE 18:2 and/or NAE 18:3 in cotyledons (a), roots (b), and seedlings (c). Beside each bar, significantly enriched GO terms are displayed in word clouds above a p‐value color scale. The size and color of the term corresponds to the relative level of enrichment and the enrichment p‐value, respectively

When the NAE 18:2 and NAE 18:3 DEGs were classified based on organ (Figures 2 and 3), the most obvious trend was a relative increase in DEGs in the organ(s) where NAE 18:2 (roots) or NAE 18:3 (cotyledons and roots) treatment induced observable growth effects. For example, while NAE 18:2 induced abundant changes in DEGs in roots, NAE 18:2 treatment led to relatively fewer DEGs in cotyledons, where its application had minimal effects on expansion or greening. NAE 18:2 and NAE 18:3 treatment led to a similar number of significantly increased transcripts in roots, from 1,682 genes and from 1,828 genes, respectively (Figure 2b). However, NAE 18:3 treatment led to relatively more genes with significantly reduced transcripts in roots (Figure 3b). Nonetheless, the majority of the genes with reduced transcripts as a result of NAE 18:2 treatment in roots also were significantly reduced by treatment with NAE 18:3. In whole seedlings, overall there were similar numbers of genes with significantly higher transcript abundances in seedlings treated with NAE 18:2 or NAE 18:3 (Figure 2c). However, there were more genes with significantly reduced transcripts in NAE 18:3 treatment in *Arabidopsis* seedlings compared with NAE 18:2 treatment (Figure 3c), similar to results with isolated roots (Figure 3b). Overall, these results suggested that there were substantial NAE‐type and organ‐specific transcriptional changes in seedlings within 1–3 hr of perception. Refer to Additional File 1 for the complete list of NAE 18:2 and NAE 18:3 DEGs.

### 3.2 Functional categories of NAE 18:2‐ and/or NAE 18:3‐responsive genes using gene ontology enrichment analysis

In order to begin understanding more about the early transcriptional changes that may play a role in directing the long‐term phenotypic changes in seedlings, the annotated function(s) of the proteins encoded by NAE 18:2 and NAE 18:3 DEGs were identified. The functional annotations of DEGs in cotyledons, roots, and seedlings were initially explored using Gene Ontology (GO) enrichment analysis. Using this method, the significantly enriched GO terms describing the biological processes, molecular functions, and cellular components were identified (Figures 2 and 3, beside histograms) using the g:Profiler web service (Reimand et al., 2016). Word clouds showing the over‐represented GO categories and the enrichment p‐values were added beside the corresponding bars in each bar graph. Refer to Additional Files 2 and 3 for the complete lists of GO categories describing NAE 18:2 and NAE 18:3 DEGs.

In cotyledons collected from NAE 18:2‐treated seedlings, some of the most significantly enriched GO terms included response to drug and multiple categories describing the transport of small molecules. Cotyledons from NAE 18:3‐treated seedlings also showed a significant enrichment in transcripts described by the GO terms response to chitin, defense response, protein serine/threonine kinase activity, and calmodulin binding. These categories suggest an increase in transcripts that encode proteins involved in the initiation of an adaptive and defense‐related signaling response in cotyledons after 1–3 hr of NAE 18:3 treatment.

Similar to cotyledons, roots collected from NAE 18:2‐ and NAE 18:3‐treated seedlings showed a significant over‐representation of transcripts described by the GO terms response to drug, response to chitin, and several additional signaling and stress response GO terms. Overall, NAE 18:2 and NAE 18:3 treatments led to a significant increase in transcripts that encode proteins involved in abiotic and biotic stress responses in roots. In cotyledons, NAE 18:2 treatment also led to a significant increase in transcripts involved in the abiotic stress response, but did not increase defense response transcripts, while NAE 18:3 treatment significantly increased both. In addition to changes identified separately in roots and cotyledons, transcriptional changes in whole seedlings were evaluated. NAE 18:2 and NAE 18:3 treatments led to a significant enrichment of transcripts described by a very similar group of GO terms in *Arabidopsis* seedlings.

In both of the organs tested, NAE 18:2 and NAE 18:3 treatments led to significant decreases in transcripts, or repression, of numerous genes (Figure 3). Cotyledons collected from NAE 18:3‐treated seedlings showed a significant decrease in transcripts involved in cell wall organization, response to auxin and xyloglucan metabolic process. A decrease in transcripts involved in these pathways would be consistent with an overall decrease in cell growth and expansion (Figures 1 and 3). NAE 18:2 and NAE 18:3 treatment also led to a significant repression of genes in roots (Figure 3b). The GO terms describing the DEGs repressed in roots by both NAE 18:2 and NAE 18:3 were similar, with those most highly enriched including cell wall organization and biogenesis and response to auxin. Significant decreases in transcripts in these GO categories were consistent with observed reductions in overall primary root length after 3 d (Figure 1).

Similar GO terms described the down‐regulated DEGs in whole seedlings treated with NAE 18:2 or NAE 18:3 (Figure 3c). In addition, the reduced transcripts in intact seedlings also showed a significant enrichment in DEGs associated with root development. Consequently, based on GO functional annotations, we conclude that the early, up‐ and down‐regulated genes induced by NAE 18:2 and NAE 18:3 treatment represented organ‐specific gene networks that ultimately lead to the observed effects of inhibition of seedling growth. These early NAE‐modulated transcripts are likely to include DEGs identified in other signal transduction pathways that modulate plant growth in response to stress, and as such, further comparisons were made to transcriptional changes described for other stress and growth pathways.

### 3.3 Comparisons of NAE‐responsive genes with hormone microarray and chitin RNA‐seq datasets

**FIGURE 4:**
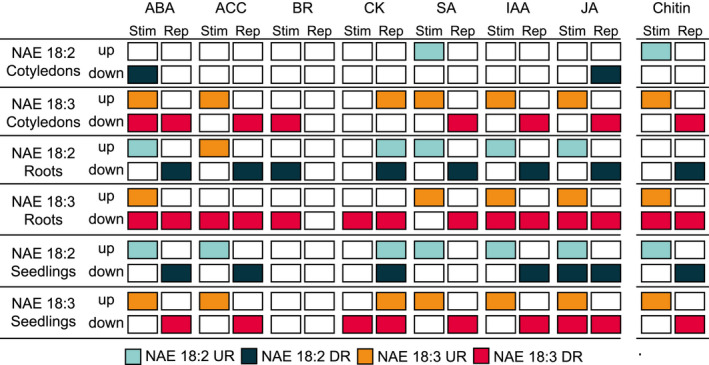
NAE 18:2 or NAE 18:3 treatment induced plant hormone‐ and chitin‐response genes. NAE 18:2 and NAE 18:3 led to an enrichment of DEGs stimulated (Stim) or repressed (Rep) by ABA, IAA, JA, SA, and chitin treatment in roots. Cotyledons treated with NAE 18:3 had an enrichment of DEGs that are stimulated (Stim) or repressed (Rep) in response to treatment with ABA, ACC, IAA, JA, and chitin. Seedlings treated with NAE 18:2 or NAE 18:3 also showed a significant overlap with ABA, ACC, IAA, JA, SA, and chitin‐response genes. NAE 18:2 and NAE 18:3 organ‐specific DEGs were compared to DEGs in hormone treatment microarray datasets (Goda et al., 2008) and a chitin RNA‐seq dataset (Stringlis et al., 2018) using the GeneOverlap program (Shen & Sinai, 2019) in R. There was no significant overlap with GA response genes. “Stimulated (Stim)/Repressed (Rep)” refers to genes up‐ or down‐regulated by hormones while “up‐regulated (up/UR)/down‐regulated (down/DR)” refers to NAE‐induced changes. ABA, abscisic acid; ACC, 1‐aminocyclopropane‐1‐carboxylic acid; BR, brassinosteroids; CK, cytokinin; DEGs, differentially expressed genes; IAA, indole‐3‐acetic acid; JA, jasmonic acid; Rep/down/DR, repressed/down‐regulated; SA, salicylic acid; Stim/up/UR, stimulated/up‐regulated

A comparison of the DEGs with hormone‐ (Goda et al., 2008) and chitin‐response genes (Stringlis et al., 2018) (both at 1 hr) led to a more in‐depth analysis of the DEGs in these pathways. First, NAE 18:2 and NAE 18:3 DEGs were compared to hormone‐induced (Stim(ulated)) and ‐Rep(ressed) genes using the GeneOverlap program (Shen & Sinai, 2019) (Additional File 4). There was a significant enrichment in ABA‐, IAA‐, JA‐, and SA‐induced and ‐repressed transcripts in roots and seedlings in response to NAE 18:2 and in cotyledons, roots, and seedlings in response to NAE 18:3 (Figure 4). These results were consistent with previously published data showing that an intact ABA signaling pathway was required to facilitate some NAE‐mediated developmental changes (Cotter et al., 2011; Keereetaweep et al., 2013, 2015; Teaster et al., 2007). In addition, the enrichment of IAA response genes was consistent with results from the GO enrichment analysis. Since JA and SA are involved in mediating biotic and abiotic stress responses in *Arabidopsis* (Ahmad et al., 2016; Lu, Greenberg, & Holuigue, 2016), this result was also consistent with the NAE‐type and organ‐specific effects described in the GO enrichment analysis for stress responses. Transcripts induced and repressed by the plant hormone ethylene also were enriched in NAE 18:2‐treated seedlings and cotyledons and seedlings treated with NAE 18:3. NAE 18:2‐ and NAE 18:3‐treatment had an opposite effect on gene expression changes when compared to brassinosteroids (BR) and cytokinin (CK) (Figure 4). For example, BR‐stimulated genes were repressed in NAE‐treated cotyledons, roots, and intact seedlings. In addition, NAE 18:2 treatment led to a gene expression trend opposite to ABA in cotyledons only.

In addition to an enrichment in hormone‐response pathways, the GO enrichment analysis also showed an over‐representation of transcripts involved in the response to chitin. All NAE 18:2‐ and NAE 18:3‐treated organs show some overlap with chitin‐response genes (Stringlis et al., 2018) (Figure 4 and Additional File 5). In particular, treatment with NAE 18:3 led to an enrichment of transcripts, some of which were induced by chitin and others which were repressed by chitin, in roots, cotyledons, and whole seedlings. Transcripts from roots of NAE 18:2‐treated seedlings showed an enrichment in genes repressed by chitin, while intact NAE 18:2‐treated seedlings showed an enrichment in transcripts that were induced or repressed by chitin treatment (Figure 4).

### 3.4 ABA‐response genes are induced or repressed by NAE 18:2 and NAE 18:3 treatment

**FIGURE 5:**
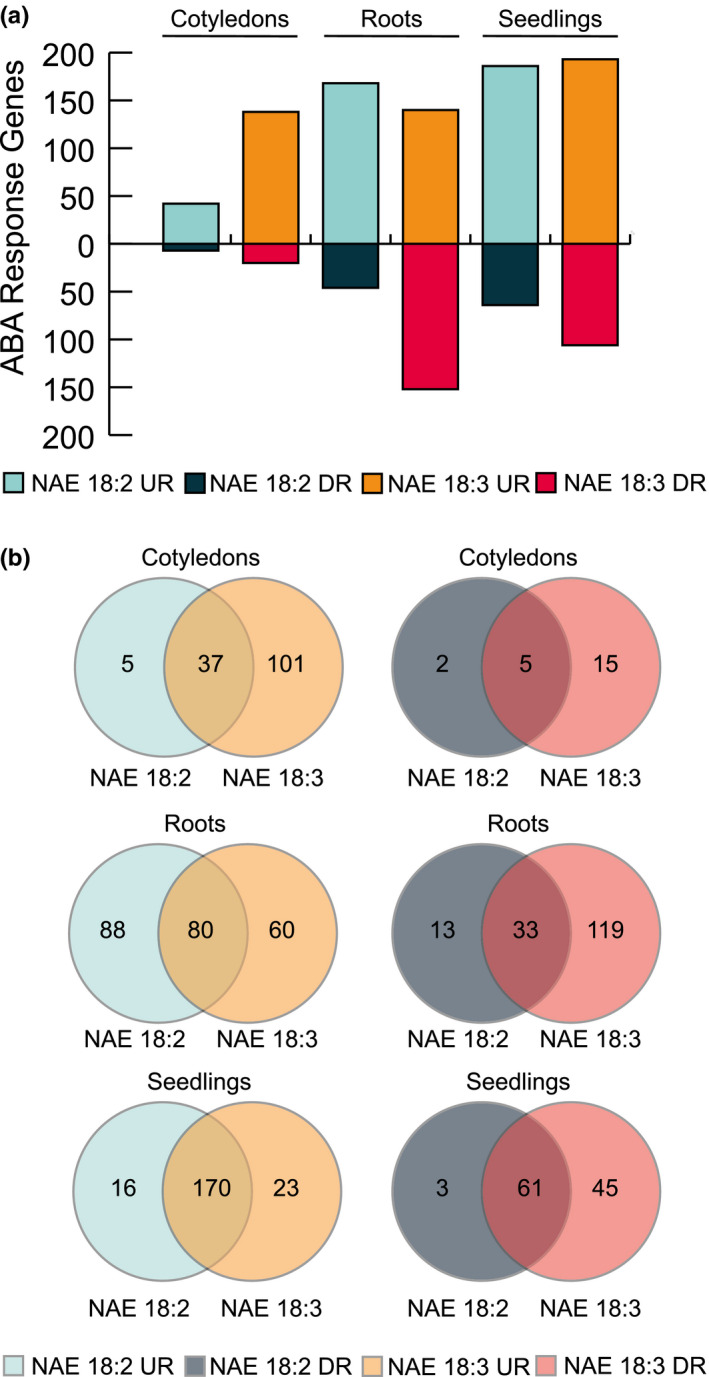
ABA‐response genes were induced or repressed by NAE 18:2 or NAE 18:3 treatment. (a) Bar graph indicating the number of ABA‐response genes induced or repressed by NAE 18:2 or NAE 18:3 in cotyledons, roots, and seedlings. (b) Venn diagrams comparing the induced or repressed ABA‐response genes in NAE 18:2‐ or NAE 18:3‐treated cotyledons, roots, and seedlings

NAE 18:2 and NAE 18:3 treatment led to transcriptome changes that shared significant overlap with changes induced by 1 hr of ABA treatment (Figures 4 and 5). In roots collected from both NAE 18:2‐ and NAE 18:3‐treated seedlings, there were 80 genes significantly induced by NAE 18:2, NAE 18:3, and ABA (Figure 5b). In addition, there were 88 ABA‐response genes specifically, significantly induced by NAE 18:2 and 60 ABA‐response genes specifically, significantly induced by NAE 18:3 in roots. Roots collected from NAE 18:2‐treated seedlings had a significant increase in ABA‐response genes that are involved in the regulation of signal transduction while roots collected from NAE 18:3‐treated seedlings had a significant increase in transcripts from ABA‐response genes involved in DNA‐binding–transcription factor activity and regulation of seedling development.

Similar to roots, cotyledons collected from NAE 18:3‐treated seedlings also showed a significant increase in transcripts from ABA‐response genes involved in DNA‐binding–transcription factor activity. In addition, cotyledons collected from NAE 18:3‐treated seedlings and roots collected from NAE 18:2‐treated seedlings had a significant increase in transcripts from ABA‐response genes involved in plant developmental processes including plant organ senescence and aging. The ABA‐response genes induced by NAE 18:2 and/or NAE 18:3 in intact seedlings were involved in the same biological processes and molecular functions as ABA‐response genes in cotyledons and roots.

Genes repressed by 1h of NAE 18:2 or NAE 18:3 treatment also shared significant overlap with genes repressed by 1 hr of ABA treatment (Figures 4 and 5a). ABA‐response genes involved in phloem transport and cell wall organization and biogenesis were significantly repressed in roots collected from NAE 18:3‐treated *Arabidopsis* seedlings. In cotyledons collected from NAE 18:2‐ and NAE 18:3‐treated seedlings, there were five ABA‐response genes significantly repressed by both of these treatments. In addition, there were 2 and 15 ABA‐response genes significantly down‐regulated in cotyledons collected from NAE 18:2‐ or NAE 18:3‐treated seedlings, respectively (Figure 5b). There were two ABA‐response transcription factors, Cytokinin‐Responsive GATA Factor 1 (CGA1) and GATA Nitrate‐Inducible Carbon‐Metabolism Involved (GNC), specifically repressed by NAE 18:3 treatment in cotyledons and seedlings. These two transcriptional regulators are involved in carbohydrate utilization and the regulation of chlorophyll biosynthetic processes.

Consistent with results from individual seedling organs, NAE 18:2 and NAE 18:3 treatment also led to the significant repression of several ABA‐responsive genes in intact *Arabidopsis* seedlings. The majority, 61 ABA‐response genes, were repressed by both NAE 18:2 and NAE 18:3 treatment (Figure 5b). However, there were 3 and 45 ABA‐response genes specifically, significantly repressed by either NAE 18:2 or NAE 18:3 treatment, respectively. The ABA‐response genes repressed by both NAE treatments were significantly enriched with genes involved in the same molecular functions and biological processes as the ABA‐response genes repressed by NAE treatments in roots and cotyledons. Refer to Additional File 7 for the complete list of GO categories describing NAE 18:2‐ and NAE 18:3‐repressed, ABA‐response genes.

### 3.5 Specific changes in genes required for ABA biosynthesis, ABA signaling, and NAE oxidation

**FIGURE 6:**
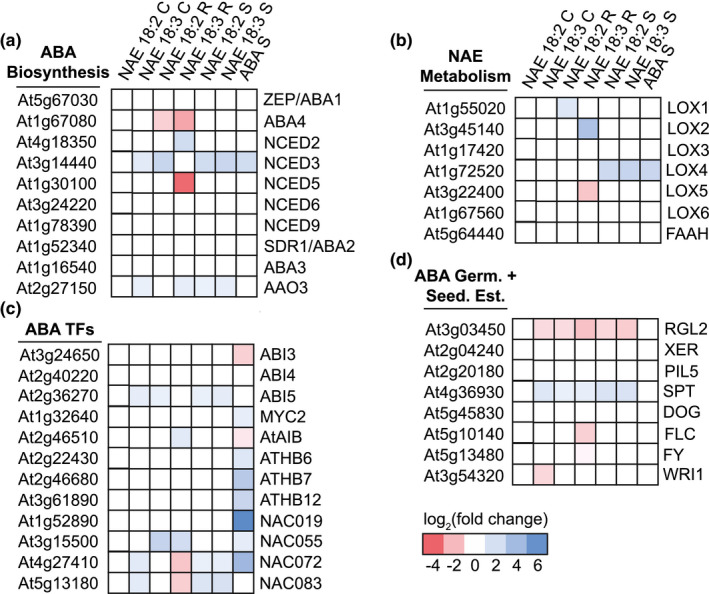
Heat maps indicating changes in ABA metabolism and signaling genes and NAE metabolic enzyme genes. (a) Gene expression changes in ABA biosynthesis enzymes after 1 hr treatment with NAE 18:2 or NAE 18:3. (b) Gene expression changes in NAE metabolic enzymes. (c) Gene expression changes in ABA‐response transcription factors in roots, cotyledons, and seedlings after 1 hr of NAE 18:2 or NAE 18:3 treatment. (d) Expression changes in genes involved in ABA‐mediated seedling germination and establishment as a result of NAE 18:2 or NAE 18:3 treatment

The log2(fold change) of ABA biosynthesis, ABA signaling, and NAE metabolic enzymes genes was compared based on organ and NAE type (Figure 6). The gene that encodes the enzyme involved in the first committed step of ABA biosynthesis, 9‐cis‐epoxycarotenoid dioxygenase 3 (NCED3) (Tan et al., 2003), was induced by NAE 18:2 in roots and intact seedlings and by NAE 18:3 in cotyledons and intact seedlings (Figure 6a). Interestingly, NCED2 was induced by NAE 18:3 in roots only. In addition, the gene that encodes the major enzyme involved in the final step of ABA biosynthesis, abscisic aldehyde oxidase 3 (AAO3) (Seo et al., 2000), also was induced in NAE 18:2‐ and NAE 18:3‐treated intact seedlings (Figure 6a). Increases in transcript levels for these genes likely are a reflection of NAE 18:2 and NAE 18:3 treatment up‐regulating the synthesis of ABA.

In previously published work, oxylipin derivatives of NAE 18:2 and NAE 18:3, namely, 9NAE‐HOD, 9‐hydroxy octadecatrienoyl ethanolamide (9NAE‐HOT), and 13‐hydroxy octadecatrienoyl ethanolamide (13NAE‐HOT) treatments also led to an increase in ABA concentration in seedlings (Keereetaweep et al., 2013, 2015). In NAE 18:2‐ and NAE 18:3‐treated *Arabidopsis* seedlings, lipoxygenase (LOX) activity may be needed in order to initiate or further promote ABA biosynthesis and signaling. Roots from seedlings treated with NAE 18:2 had a significant increase in LOX1 transcripts while roots from seedlings treated with NAE 18:3 had a significant increase in LOX2 transcripts (Figure 6b). In addition, transcripts of the LOX4 gene were significantly increased by NAE 18:2, NAE 18:3, and ABA treatment in *Arabidopsis* seedlings (Figure 6b).

Since several ABA‐response genes are significantly up‐ or down‐regulated after 1 hr of treatment with NAE 18:2 or NAE 18:3, the transcript abundance of transcription factors involved in modulating the expression of genes in response to ABA also might have been changed. ABI5, a bZIP transcription factor, was induced by NAE 18:2 in roots and seedlings and by NAE 18:3 in cotyledons and seedlings (Figure 6c). In addition, a few ABA‐responsive, NAC transcription factors were also induced by NAE 18:2 and NAE 18:3 in roots, cotyledons, and/or seedlings (Figure 6c). The abundance of transcripts from genes involved in germination and seedling establishment processes that are modulated by ABA (Finkelstein, 2013) was also analyzed (Figure 6d). Some of these genes are significantly repressed in NAE 18:2‐ and NAE 18:3‐treated organs. However, the gene encoding the bHLH transcription factor, SPATULA (SPT), is induced by NAE 18:3 in all roots, cotyledons, and seedlings and by NAE 18:2 in roots and seedlings. Taken altogether, transcriptional programs induced by NAE 18:2 and NAE 18:3 within 1–3 hr are consistent with ABA and NAE interaction during early seedling development as previously suggested (Blancaflor et al., 2014; Keereetaweep et al., 2013, 2015), and here this is resolved at early time points and at the level of NAE‐type and organ‐specific transcriptional changes.

### 3.6 Chitin‐response genes are induced by NAE 18:2 and NAE 18:3 treatment

**FIGURE 7:**
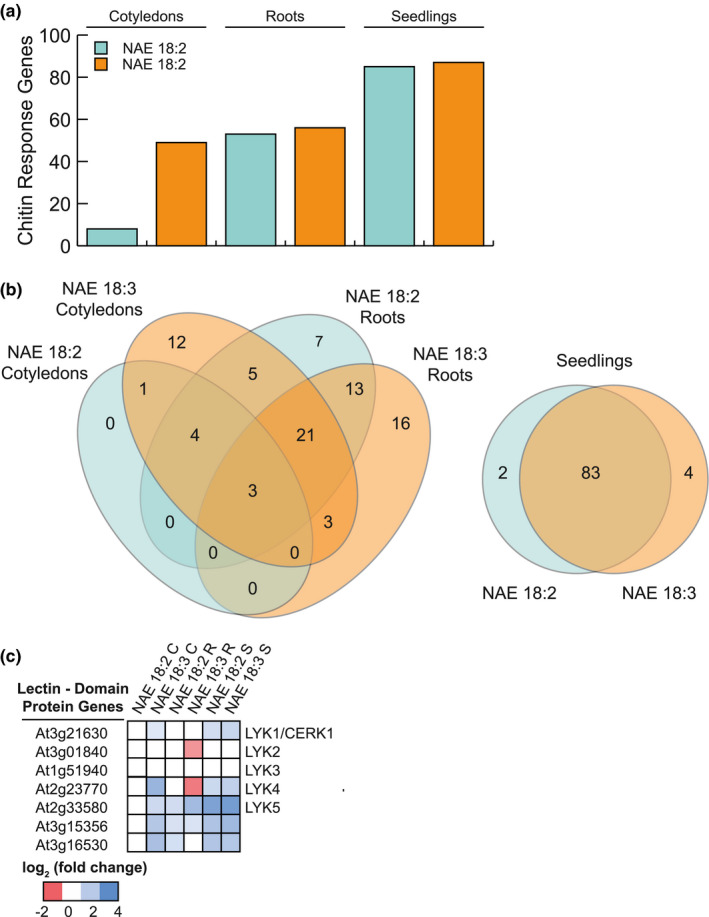
NAE 18:2 and NAE 18:3 led to a significant enrichment in chitin‐response genes. (a) Bar graph indicating the number of chitin‐response genes induced by NAE 18:2 or NAE 18:3 in cotyledons, roots, and seedlings. (b) Venn diagrams comparing chitin‐response genes in cotyledons and roots collected from NAE 18:2‐ and NAE 18:3‐treated seedlings (left) and comparing chitin‐response genes in seedlings (right). (c) Heat map showing the log2(fold change) of Lectin‐domain containing protein genes in NAE 18:2‐ and NAE 18:3‐treated cotyledons (C), roots (R), and seedlings (S)

NAE 18:2 and NAE 18:3 treatment also led to an increase in transcripts previously shown to be induced by chitin treatment (Figures 4 and 7a). In order to determine if there were NAE‐type and organ‐specific aspects to this enrichment, the DEGs in the response to chitin GO category from root and cotyledon samples were compared. Refer to Additional File 8 for the complete list of chitin‐response genes induced by NAE 18:2 and/or NAE 18:3. In NAE 18:2‐treated seedlings, transcripts were significantly increased for 8 chitin‐response genes in cotyledons and 53 in roots (Figure 6a,b). There were 49 and 56 chitin‐response genes significantly up‐regulated by NAE 18:3 treatment in cotyledons and roots, respectively (Figure 7a,b). When these DEGs were compared, there were 24 DEGs common to roots from NAE 18:2‐treated seedlings and cotyledons and roots from NAE 18:3‐treated seedlings (Figure 7b). Within these 24 common DEGs, there were transcripts that encoded 16 transcription factors, 5 ubiquitin ligases, 2 Lectin‐domain containing proteins, and a putative sucrose transporter, Early Response to Dehydration 6 (ERD6). The 16 significantly, up‐regulated transcription factors belong to several *Arabidopsis* gene families, but the most highly represented group was the Zinc Finger OF *Arabidopsis* thaliana (ZAT) group of the C2H2‐type, plant‐specific family of transcription factors. The two Lectin‐domain containing protein genes were LysM‐containing Receptor‐Like Kinase (RLK) 5 (LYK5) and AT3G15356 (Figure 7c).

Roots collected from NAE 18:2‐ and NAE 18:3‐treated seedlings shared an additional 13 chitin‐response genes that were not in cotyledons treated with NAE 18:3 (Figure 7b). Among these 13 DEGs, nine transcripts encoded transcription factors and four encoded ubiquitin ligases. Roots from NAE 18:2‐treated seedlings and cotyledons from NAE 18:3‐treated seedlings shared nine chitin‐response genes including six transcription factors, a RING/U‐box ubiquitin ligase (AT5G38895), the Receptor Like Protein 52 (RLP52), and a Lectin‐domain containing protein gene (AT3G16530) (Figure 7c).

NAE 18:2 and NAE 18:3 treatment also led to a significant increase in transcripts from chitin‐response genes that were NAE type and organ specific (Figure 7b). For example, NAE 18:2 treatment led to an increase in transcripts in roots encoding six transcription factors and the regulatory protein gene, Non‐Expressor of Pathogenesis Related Genes 2 (NPR2). NAE 18:3 treatment led to an increase in transcripts in roots encoding 10 transcription factors, five ubiquitin ligases, and the serine/threonine‐protein kinase, AVRPPHB Susceptible 1 (PBS1)‐ Like 27 (PBL27). NAE 18:3 treatment also induced the expression of 13 unique chitin‐response genes specifically in cotyledons including six transcription factors and one ubiquitin ligase. In addition, transcripts of two Lectin RLKs, LYK4 and Chitin Elicitor Receptor Kinase 1 (CERK1) (Figure 7c), a Leucine‐Rich Repeat (LRR)‐RLK, Impaired Oomycete Susceptibility 1 (IOS1), and the Somatic Embryogenesis Receptor Kinase 4 (SERK4) were induced specifically in cotyledons by NAE 18:3.

Consistent with results from individual seedling organs, in intact *Arabidopsis* seedlings treated with NAE 18:2 or NAE 18:3, there was a significant increase in transcripts from 85 and 87 chitin‐response genes, respectively (Figure 7a,b). When these transcripts were compared, 83 were common to NAE 18:2‐ and NAE 18:3‐treated seedlings (Figure 7b). Within these 83 DEGs, 54 encoded transcription factors, 15 encoded ubiquitin ligases, and 5 encoded Lectin‐domain containing proteins (Figure 7c). In addition, 72 of the 83 common DEGs also were up‐regulated in cotyledons and/or roots from NAE 18:2‐ or NAE 18:3‐treated seedlings. It seems that transcriptional programs activated by NAE in seedlings overlap substantially with those induced by chitin, and at these early time points there are some that are organ‐specific and some that are NAE‐type specific. These early NAE‐/chitin‐responsive genes represent candidates for future follow‐ups as potential direct and indirect targets of NAE action.

### 3.7 Numerous transcription factors are induced or repressed by NAE 18:2 and NAE 18:3 treatment

**FIGURE 8:**
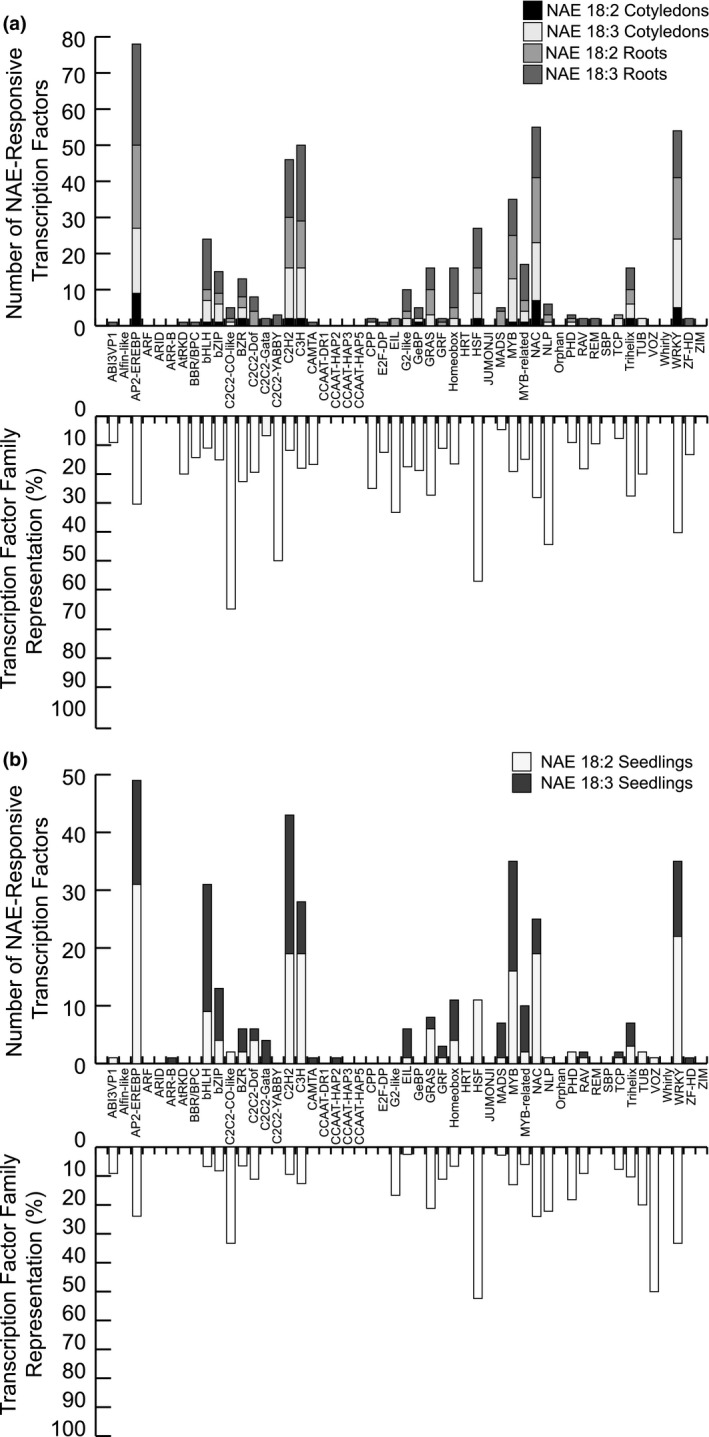
Transcription factors induced by NAE 18:2 and/or NAE 18:3 in cotyledons, roots, and seedlings. (a) Bar graph(s) indicating the number of transcription factor genes and the percentage of genes represented in each transcription factor family that are induced by NAE 18:2 and/or NAE 18:3 in cotyledons and/or roots belonging to each *Arabidopsis* transcription factor gene family. (b) Bar graph(s) indicating the number of transcription factor genes and the percentage of genes represented in each transcription factor family that are induced by NAE 18:2 and/or NAE 18:3 in seedlings belonging to each *Arabidopsis* transcription factor gene family

**FIGURE 9:**
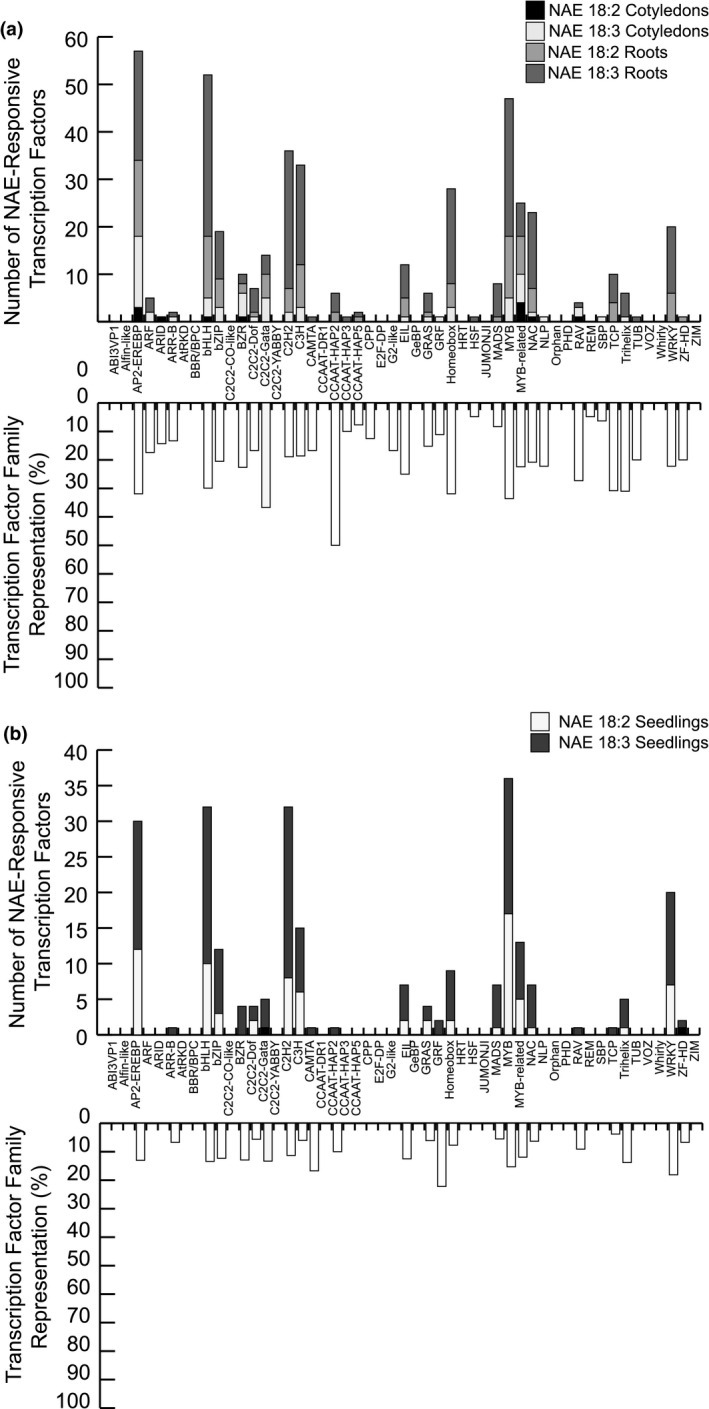
Transcription factors repressed by NAE 18:2 and/or NAE 18:3 in cotyledons, roots, and seedlings. (a) Bar graph(s) indicating the number of transcription factor genes and the percentage of genes represented in each transcription factor family that are repressed by NAE 18:2 and/or NAE 18:3 in cotyledons and/or roots belonging to each *Arabidopsis* transcription factor gene family. (b) Bar graph(s) indicating the number of transcription factor genes and the percentage of genes represented in each transcription factor family that are repressed by NAE 18:2 and/or NAE 18:3 in seedlings belonging to each *Arabidopsis* transcription factor gene family

There were numerous transcription factor genes significantly induced or repressed by NAE 18:2 and/or NAE 18:3 treatment and these were analyzed in greater detail (Figures 8a,b and 9a,b). In roots and cotyledons collected from NAE‐treated seedlings, there were NAE‐type and organ‐specific, induced, and repressed transcription factor genes. The NAE 18:2 and NAE 18:3 induced and repressed transcription factor genes were classified based on family (Figures 8a,b and 9a,b). There were members of 39 gene families induced by NAE 18:2 and/or NAE 18:3 treatment. The most highly represented families induced by NAE 18:2 and/or NAE 18:3 treatment included C2C2‐CO‐like, C2C2‐YABBY, HSF, NLP, and WRKY transcription factor families (Figure 8a,b). NAE 18:2 and NAE 18:3 treatment also led to the repression of transcription factors belonging to 32 different gene families. The most highly repressed transcription factor gene families included AP2‐EREBP, bHLH, C2C2‐GATA, CCAAT‐HAP2, Homeobox, and MYB transcription factor families (Figure 9a,b). Overall, the families of transcription factors significantly up‐ and down‐regulated by NAEs in dissected organs (Figures 8a and 9a) and intact seedlings (Figures 8b and 9b), and the processes in which they are involved, were consistent with the downstream physiological changes in seedling growth and development induced by NAEs. These specific transcription factors represent a rich source of genes to explore in the future for NAE‐responsive transcriptional regulatory networks.

### 3.8 NAE‐type and organ‐specific decreases in transcripts involved in growth and expansion and chlorophyll biosynthesis

**FIGURE 10:**
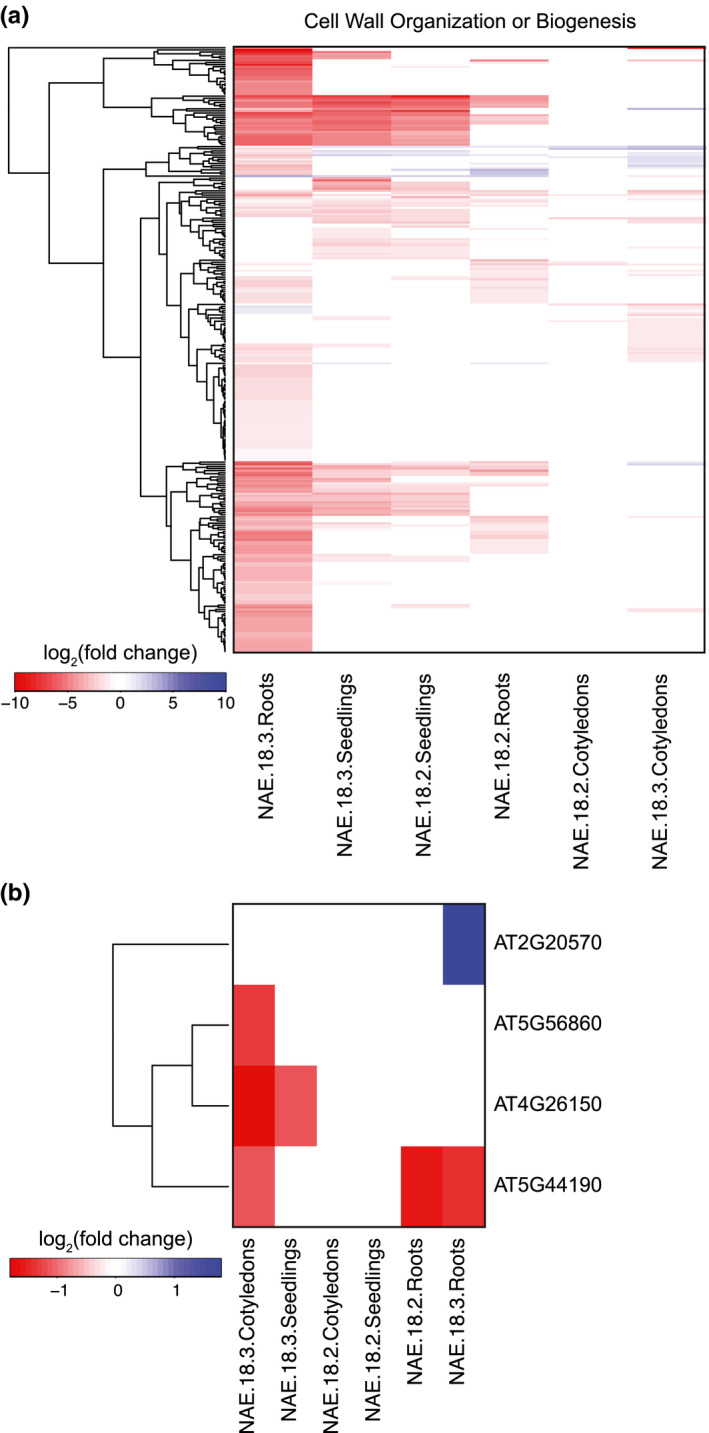
Heat maps indicating transcript abundance changes in cell wall genes and chlorophyll transcription factor genes. (a) Heat map showing the log2(fold change) of cell wall organization or biogenesis genes modulated by NAE 18:2 or NAE 18:3 treatment in cotyledons, roots, and seedlings. (b) Heat map showing the log2(fold change) of transcriptional regulators of chlorophyll biosynthesis in NAE 18:2‐ or NAE 18:3‐treated cotyledons, roots, and seedlings

When *Arabidopsis* seedlings were treated with NAE 18:2 or NAE 18:3, there was a significant decrease in primary root elongation or overall seedling growth, respectively, over a several day period (Figure 1 and [Keereetaweep et al., 2013, 2015]). In order to determine what molecular changes facilitated these decreases in growth, functional annotations were used to identify groups of genes that are involved in plant cell growth and expansion. In *Arabidopsis* seedlings, treatment with NAE 18:2 or NAE 18:3 led to a decrease in transcripts that encode proteins involved in cell wall organization or biogenesis in an NAE‐type and organ‐specific manner (Figure 10). NAE 18:3 treatment reduced the abundance of these transcripts in both cotyledons and roots, while NAE 18:2 treatment led to a decrease in transcripts from these genes primarily in roots (Figure 10a). Several of the proteins encoded by these transcripts are involved in primary cell wall or secondary cell wall biosynthesis.

For example, NAE 18:3 treatment led to a significant decrease in abundance of four cellulose synthase genes in roots or cotyledons. Transcripts that encode the proteins, Cellulose Synthase Catalytic Subunit A 7 (CESA7), CESA4, and CESA8 were significantly decreased in roots, and transcripts that encode CESA5 were significantly less abundant in cotyledons. CESA7 transcripts were also significantly less abundant in *Arabidopsis* seedlings treated with NAE 18:2. NAE 18:3 treatment also led to a significant decrease in transcripts that encode STELLO1 (STL1), a modulator of cellulose synthase complex assembly and trafficking (Zhang et al., 2016). Refer to Additional File 11 for a complete list of cell wall organization or biogenesis genes that are repressed by NAE 18:2 and/or NAE 18:3 treatment.

In *Arabidopsis* seedlings treated with NAE 18:2 or NAE 18:3, there also were significant decreases in the abundance of transcripts that encode enzymes involved in xyloglucan metabolism including xyloglucan endotransglycosylases/hydrolases (XTHs), xyloglucan endotransglucosylases/hydrolases (XETs), and a xyloglucan‐specific galacturonosyl transferase (XUT). In addition, transcripts that encode pectin metabolic enzymes and inhibitors also were significantly repressed, including pectin esterases, members of the pectin lyase‐like superfamily, and pectin methylesterase inhibitors. NAE 18:2 and NAE 18:3 treatment also led to a significant decrease in fasciclin‐like arabinogalactan proteins, cell wall proteins involved in cell adhesion (Johnson, Jones, Bacic, & Schultz, 2003).

NAE 18:2 and NAE 18:3 treatment also led to a significant decrease in abundance of a significant number of transcripts that encode structural components of cell walls. Transcripts that encode several extensin‐like family proteins, expansins, and additional cell wall structural proteins were significantly decreased as a result of NAE 18:2 and NAE 18:3 treatment. Overall, the number of significantly repressed cell wall biogenesis or organization and cell wall structural components genes was relatively higher in organs where NAE 18:2 and/or NAE 18:3 treatment led to a decrease in cell expansion and growth (Figure 10a).

NAE 18:3 treatment also significantly decreased the abundance of transcripts that encode transcription factors involved in promoting chloroplast biogenesis (Chiang et al., 2012; Waters, Moylan, & Langdale, 2008). GNC and CGA1 were significantly repressed in cotyledons and seedlings treated with NAE 18:3 (Figure 10b). Golden2‐Like 2 (GLK2) was also significantly repressed in roots of NAE 18:2 seedlings and cotyledons and roots of NAE 18:3‐treated seedlings. Taken together, identified decreases in transcripts that encode proteins involved in cell wall biogenesis and organization and chlorophyll biosynthesis most likely contribute to the NAE 18:2‐ and NAE 18:3‐mediated phenotypic changes in seedling growth and development.

## 4 DISCUSSION

Exogenous treatment with the two most abundant polyunsaturated NAEs in *Arabidopsis* seeds, NAE 18:2 and NAE 18:3, induced organ‐specific phenotypic changes in *Arabidopsis* seedlings (Keereetaweep et al., 2013, 2015) that are characteristic of the ABA‐mediated process called secondary dormancy (Lopez‐Molina et al., 2001). In the case of NAE 18:2, primary root elongation was inhibited by an ABA‐dependent process (Keereetaweep et al., 2015). Alternatively, the exogenous application of NAE 18:3 led to a decrease in cotyledon expansion, increase in chlorophyll breakdown, and an overall decrease in seedling growth (Keereetaweep et al., 2013). The bifurcating nature of this pathway leads to questions about the spatial production of NAEs, perception of NAEs, and the signaling mechanisms involved in mediating the NAE 18:2 and NAE 18:3 organ‐specific responses. Here, RNA‐seq analysis helped to gain insight into the organ‐specific molecular responses in *Arabidopsis* seedlings to exogenous treatment with NAE 18:2 or NAE 18:3.

**FIGURE 11:**
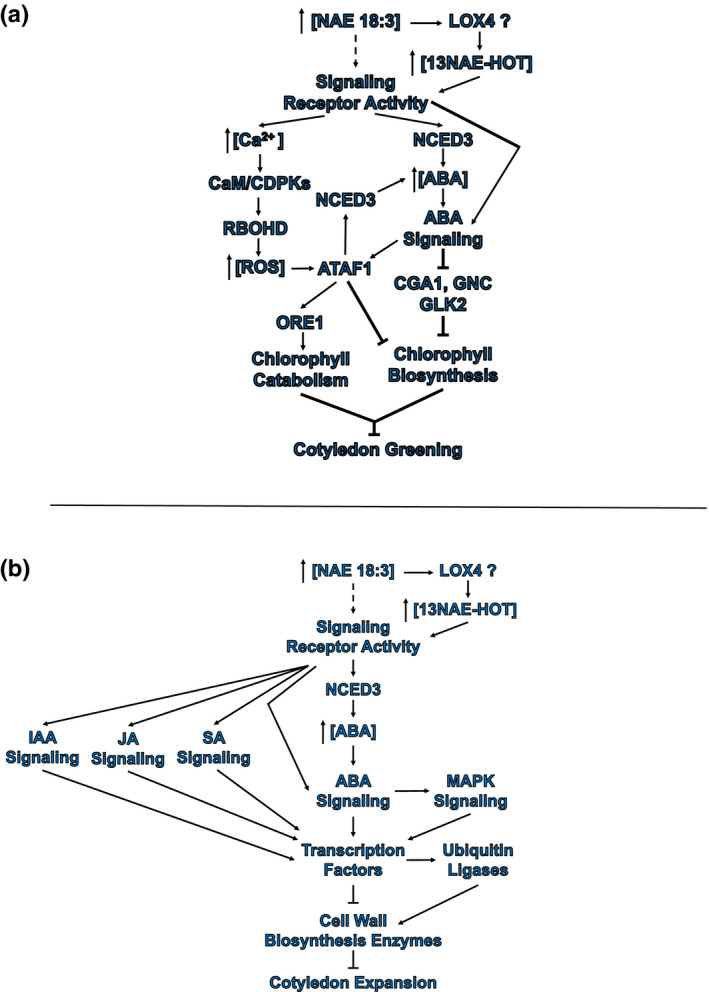
Hypothetical models of the early transcriptional changes in cotyledons as a result of NAE 18:3. Hypothetical model of the early transcriptional changes in cotyledons that leads to chlorophyll breakdown (a) or a decrease in cotyledon expansion (b). NAE 18:3 may be bound to a signaling receptor or be oxidized by lipoxygenase (LOX) 4. LOX4 introduces a hydro(per)oxy group at the 13 position on the acyl chain of NAE 18:3 generating 13NAE‐H(P)OT. The NAE oxylipin, 13NAE‐HOT, may bind to a signaling receptor that upon activation, indirectly modulates the abundance of transcripts that encode proteins involved in several plant hormone response or stress response pathways. In addition, transcripts that encode enzymes in ABA biosynthesis increase in abundance. The activation of the signaling cascade increases or decreases the abundance of transcription factors and ubiquitin ligases. (a) The change in abundance of transcription factors is presumed to inhibit the expression of CGA1, GNC, and GLK2. In addition, ubiquitin ligases may facilitate the turnover of proteins involved in cotyledon greening. Ultimately, there is a decrease in chlorophyll content. In addition, the presumed activation of a signaling receptor by NAE 18:3 and/or 13NAE‐HOT results in an increase in transcripts involved in calcium and ROS signaling. Transcripts that encode the ATAF1 transcription factor increase in abundance. The ATAF1 transcription factor contributes to the increase in ABA biosynthesis by activating the expression of ABA biosynthesis enzyme(s). ATAF1 also represses chlorophyll biosynthesis and activates chlorophyll catabolism by activating or repressing genes involved in these pathways. (b) The change in abundance of transcription factors inhibits the expression of cell wall biosynthesis enzymes and structural components of the cell walls. In addition, ubiquitin ligases facilitate the turnover of proteins involved in cell expansion. Ultimately, these pathways lead to a reduction in cotyledon greening and expansion

In developing seedlings, cotyledon greening was reversed and expansion was inhibited as a result of exogenous treatment with NAE 18:3. This RNA‐seq analysis showed that these developmental changes were preceded by NAE 18:3‐ and cotyledon‐specific transcriptome changes including changes in transcript abundance of molecular components from several, well‐studied signaling pathways (Figure 11). As a result of NAE 18:3 treatment, transcripts that encode LOX 4 enzymes significantly increased in *Arabidopsis* seedlings (Figures 6 and 11). These enzymes are involved in converting NAE 18:3 into 13NAE‐HOT, an oxylipin derivative (Keereetaweep et al., 2013). After NAE 18:3 treatment, NAE 18:3 and/or 13NAE‐HOT presumably bound to and activated a signaling receptor. The activation of a NAE 18:3 and/or 13NAE‐HOT receptor could have indirectly initiated the transcription of or stabilized transcripts that encode ABA biosynthesis and ABA signaling components (Figures 5 and 6). In addition, IAA, JA, and SA signaling components were induced or repressed by the activation of the NAE 18:3 signaling pathway in cotyledons (Figure 4). Individual and/or networks of NAE 18:3 signaling components inhibited chlorophyll biosynthesis and activated chlorophyll catabolism leading to cotyledon de‐greening (Figures 10 and 11a). In addition, NAE 18:3 treatment also led to a decrease in cotyledon expansion. The activation of a NAE 18:3 or 13NAE‐HOT signaling receptor could have also indirectly initiated or contributed to a decrease in cotyledon expansion by modulating the abundance of enzymes involved in cell growth and expansion and the components necessary for these processes leading to a decrease in cotyledon expansion and growth (Figure 11b).

**FIGURE 12:**
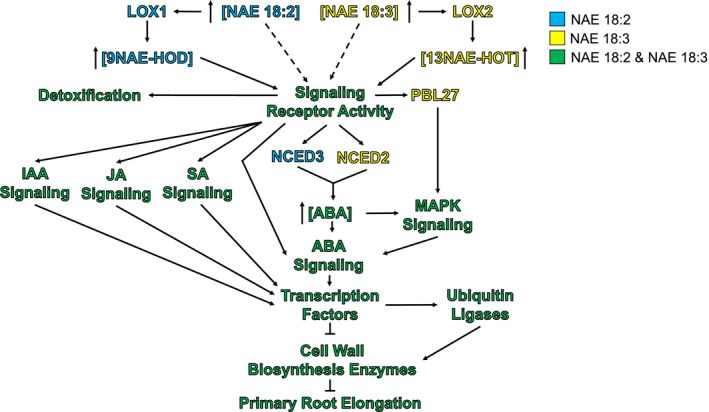
Hypothetical model of the early transcriptional changes in roots to NAE 18:2 and NAE 18:3. NAE 18:2 may be bound to a signaling receptor or oxidized by lipoxygenase (LOX) 1. LOX 1 introduces a hydro(per)oxy group at the 9 position in the acyl chain of NAE18:2 generating 9NAE‐H(P)OD. NAE 18:3 may be bound to a signaling receptor or be oxidized by LOX 2. LOX 2 introduces a hydro(per)oxy group at the 13 position in the acyl chain of NAE18:3 generating 13NAE‐H(P)OT. (a and b) The NAE oxylipins, 9NAE‐HOD or 13NAE‐HOT, are presumed to bind to a signaling receptor that upon activation modulates the abundance of transcripts that encode proteins involved in several plant hormone response or stress response pathways. NAE 18:3 or 13NAE‐HOT increased the abundance of PBL27, a chitin signaling component that links receptor activation to MAPK signaling specifically in roots. In addition, transcripts that encode enzymes in the ABA biosynthesis pathway or MAPKs increased in abundance. The activation of the signaling receptor and/or downstream secondary messengers increases or decreases the abundance of transcription factors and ubiquitin ligases. The change in abundance of transcription factors inhibits the expression of cell wall biosynthesis enzymes and structural components of the cell walls. In addition, ubiquitin ligases facilitate the turnover of proteins involved in primary root elongation. Ultimately, these pathways lead to a reduction in primary root elongation

By contrast, exogenous treatment with NAE 18:2 or NAE 18:3 led to primary root growth inhibition. Although both of these NAE treatments led to the same visible changes in roots, some of the transcriptome changes that preceded this change were NAE specific (Figure 12). NAE 18:2 and NAE 18:3 treatment led to an increase in the abundance of transcripts of the encoded LOX enzymes (Figures 6 and 12). These two NAEs, or the NAE‐oxylipins generated by the LOX enzymes, presumably bound to and activated a signaling receptor. These activated receptor(s) could have initiated or contributed to the NAE‐signaling responses by indirectly increasing or decreasing the abundance of transcripts that encode proteins involved in several signaling pathways (Figure 12). In addition, ABA biosynthesis could have been activated by the proteins encoded by the NAE‐specific NCED transcripts (Figures 6 and 12). NAE‐specific and/or organ‐specific DEG(s) initiated or contributed to a decrease in growth by modulating the abundance of cell wall components and enzymes involved in cell growth leading to a decrease in primary root elongation (Figure 12).

### 4.1 Genes targeted by NAE 18:2 and/or NAE 18:3

During seedling establishment, NAE 18:2 and NAE 18:3 were reported to be converted into the corresponding NAE‐oxylipins by LOX enzymes, and it was the NAE oxylipins that possessed the actual growth inhibitory activity (Blancaflor et al., 2014; Keereetaweep et al., 2013, 2015; Kilaru et al., 2011). Several LOX transcripts were modulated by NAE 18:2 and/or NAE 18:3 (Figure 6b). NAE 18:2 treatment significantly increased LOX1 transcripts in roots and LOX4 transcripts in seedlings, while NAE 18:3 treatment significantly increased LOX2 transcripts in roots and LOX4 transcripts in seedlings. LOX1 is a 9S‐lipoxygenase (Bannenberg, Martínez, Hamberg, & Castresana, 2009) that catalyzes the oxygenation of NAE 18:2 to 9NAE‐HOD and NAE 18:3 to 9NAE‐HOT (Keereetaweep et al., 2013, 2015; Kilaru et al., 2011). LOX 2 and LOX4 are 13S‐lipoxygenases that selectively oxygenated linolenic acid (Bannenberg et al., 2009), the acyl chain of NAE 18:3. Using *lox* mutants, previous studies have shown that the NAE 18:2‐derived oxylipin, 9NAE‐HOD, arrested root growth, and both 9NAE‐HOT and 13NAE‐HOT disrupted chloroplast development (Keereetaweep et al., 2013, 2015). In this study, the NAE‐type and organ‐specific increases and decreases in LOX transcripts suggest that there may be a spatial difference in the organ‐specific accumulation of 9‐LOX‐ or 13‐LOX‐derived NAE oxylipin species that precedes the downstream phenotypic changes (Figures 6 and 12).

Previous studies by Keereetaweep et al. (2015) showed that NAE 18:2 modulates primary root elongation through an ABA receptor–mediated process. In addition, exogenous treatment, with NAE 18:2 or NAE 18:3, led to an increase in the ABA content of *Arabidopsis* seedlings (Keereetaweep et al., 2013, 2015), suggesting that NAE 18:2, NAE 18:3, and ABA may synergistically modulate the expression of the same genes involved in secondary dormancy. Indeed, RNA‐seq analysis showed that there was a significant enrichment in ABA‐response genes that were induced or repressed by NAE 18:2 and/or NAE 18:3 treatment (Figures 5, 6, 11 and 12). In both roots and cotyledons, the majority of ABA‐response genes were modulated in an NAE‐type and organ‐specific way (Figure 5b). For example, in roots, NAE 18:2 treatment led to an increase in transcripts of three 2C‐type Protein Phosphatases (PP2Cs), including Highly ABA‐Induced PP2C Gene 1 (HAI1), HAI2, ABA Insensitive 2 (ABI2), and the SNF1‐related Protein Kinase 2 (SnRK2), Open Stomata 1 (OST1), and two MAPKs, MAPKKK17 and MAPKKK18. However, NAE 18:3 treatment led to an increase in HAI2 and MAPKKK18, in roots, but led to an increase in HAI2, ABI2, and MAPKKK18 in cotyledons.

In addition, although a significant number of ABA‐response genes were induced or repressed by NAE 18:2 or NAE 18:3, the expression of most ABA biosynthesis genes and ABA transcription factors was not significantly changed after 1–3 hr of NAE 18:2 or NAE 18:3 treatment (Figure 6a,c). This suggests that the 1–3 hr time point has captured events prior to the full activation of the ABA response; nevertheless, transcript levels of 9‐cis‐epoxycarotenoid dioxygenases (NCED2, NCED3), the enzyme involved in the first committed step in ABA synthesis, were significantly increased by NAE 18:2 and NAE 18:3 (Figure 6a). Interestingly, NCED3 was induced by NAE18:2, while NCED2 was induced by NAE18:3 in roots only, suggesting that there may be some spatial differences in the induction of ABA synthesis by these two different NAEs in roots and cotyledons. This significant overlap of genes induced and repressed by ABA, NAE 18:2, and NAE 18:3 supports the hypothesis that these three compounds modulate the expression of the same genetic pathways that lead to the reduced growth accompanied with secondary dormancy. In addition, the lipid‐type and organ‐specific expression trends of these genes suggest that distinct networks may lead to the observed differences in downstream phenotypic changes by these two different NAEs.

In addition to overlap with ABA signaling, NAE 18:2 and NAE 18:3 treatment also led to an increase and decrease in transcripts that share significant overlap with genes that are differentially expressed as a result of IAA, JA, and SA treatment (Figures 4, 11, and 12). Several of the genes that overlap with the hormone response pathways are components of the hormone signaling pathways or transcription factors activated by these pathways. When seedlings were treated with NAE 18:2 or NAE 18:3, transcripts from some Aux/IAA and/or Small Auxin Up‐regulated RNA (SAUR) genes were significantly increased. In addition, transcripts from calcium induced protein genes, such as Calmodulin 38 (CaM38), were significantly increased by NAE 18:2 and NAE 18:3 and transcripts from Respiratory Burst Oxidase Homologue D (RBOHD), were specifically significantly increased by NAE 18:2 in roots. These common and NAE‐type gene expression changes that overlap with hormone response pathways suggest that the gene networks modulated by NAE 18:2 and NAE 18:3 may intersect with multiple plant hormone pathways during growth inhibition that is characteristic of secondary dormancy in seedlings.

Our RNA‐seq analyses also showed that NAE 18:2 and NAE 18:3 treatment led to increases in transcripts that were also significantly increased by chitin treatment (Stringlis et al., 2018) (Figure 7) in the organs where treatment with NAE 18:2 (roots) and NAE 18:3 (roots and cotyledons) led to phenotypic changes. Upon closer inspection, multiple Lectin‐RLKs and Lectin‐domain containing proteins were induced in response to NAE 18:2 or NAE 18:3 treatment (Figure 7c). In addition, a common group of chitin‐response genes, including transcription factors and ubiquitin ligases, were induced in roots of NAE 18:2‐treated seedlings and roots and cotyledons of NAE 18:3‐treated seedlings. Chitin signaling components, including the receptor‐like cytoplasmic kinase, PBL27 (Yamada et al., 2016), were induced in a lipid‐type and organ‐specific manner (Figures 11 and 12).

Although chitin and NAEs are not structurally similar, receptors with extracellular Lectin domains have been shown to bind the microbial molecule, nodulation (Nod) factor (Limpens et al., 2003; Madsen et al., 2003; Radutoiu et al., 2003). Nod factors are lipochitooligosaccharides that contain a chitin oligomeric backbone linked to an acyl chain. NAE 18:2 and NAE 18:3, or metabolites thereof, may increase the expression of chitin‐response genes by interacting with *Arabidopsis* Lectin‐RLKs. Alternatively, NAE 18:2 and NAE 18:3 treatment may increase the expression of chitin‐response genes through a receptor that specifically recognizes NAE molecules. The latter case may be supported by a recent study showing that NAE 12:0 produced by the fungus, *V. dahliae*, led to changes similar to the phenotypic changes induced by the exogenous application of NAEs (Zhang et al., 2019). Based on these results, it is possible that plants contain an NAE or NAE oxylipin perception mechanism that is activated by NAE 18:2 and/or NAE 18:3, and upon activation, this perception mechanism may induce the expression of chitin/fungal defense‐response genes. In any case, exogenous treatment with NAE 18:2 and NAE 18:3 led to an increase in transcript abundance of Lectin‐RLKs, Lectin‐domain containing proteins, and numerous chitin‐response genes (Figure 7). This significant enrichment of transcripts from chitin‐signaling component genes and chitin‐response genes as a result of NAE 18:2 and NAE 18:3 treatment suggests that these receptors and signaling components are somehow involved in modulating the downstream NAE‐mediated phenotypic changes in seedling growth and development.

In this study, it was not surprising that many of the early transcriptome changes were involved in reprogramming gene expression, since *Arabidopsis* seedlings were collected after 1–3 hr of NAE 18:2 or NAE 18:3 treatment. In particular, NAE 18:2 and NAE 18:3 treatment significantly increased or decreased the transcript level of several transcription factor genes (Figures 8, 9, 11, and 12). These transcription factor genes were modulated in a lipid‐type and organ‐specific manner (Figures 8 and 9). Collectively, the gene expression trends and functional annotations of the transcription factor genes up‐ and down‐regulated by NAE 18:2 and/or NAE 18:3 support the hypothesis that some of the early transcriptome changes captured in this study are involved in reprogramming gene expression. This transcriptome reprogramming likely initiates the processes involved in the downstream phenotypic changes induced by exogenous treatment with NAE 18:2 or NAE 18:3, and these transcription factors represent potential key regulatory components for further examination.

### 4.2 Genes involved in cotyledon de‐greening

As a result of NAE 18:3 treatment, there was a decrease in abundance of two GATA transcription factors, GNC and CGA1, specifically in cotyledons (and seedlings) treated with NAE 18:3 (Figures 10 and 11). These two transcription factors promoted chloroplast development, growth, and division (Chiang et al., 2012). In addition, the abundance of transcripts that encode the NAC transcription factor, *Arabidopsis* thaliana Activation Factor 1 (ATAF1), significantly increased in cotyledons after 1 hr of NAE 18:3 treatment. The ATAF1 transcription factor has been shown to activate the expression of another transcription factor, Oresara 1 (ORE1), and repress the expression of the transcription factor, Golden2‐Like 1 (GLK1) (Garapati, Xue, Munné‐Bosch, & Balazadeh, 2015). ORE1 is a positive regulator of senescence that directly and indirectly activates several Senescence Activating Genes (SAGs) (Qiu et al., 2015). GLK1 and GLK2 play key roles in chloroplast development in *Arabidopsis* (Garapati et al., 2015; Waters et al., 2009). A decrease in transcripts that encode CGA1, GNC, and GLK2, and an increase in transcripts that encode ORE1, would support a decrease in chlorophyll biosynthesis and chloroplast development and an increase in SAGs and chlorophyll catabolism (Figure 11b), changes associated with the de‐greening observed in NAE 18:3‐treated seedlings.

### 4.3 Genes involved in growth: cotyledon expansion and growth and primary root elongation

In roots of NAE 18:2‐ and NAE 18:3‐treated seedlings, there was a significant decrease in transcripts that encode transcriptional regulators of secondary cell wall biosynthesis. In particular, NAE 18:2 and NAE 18:3 treatment deceased the abundance of Vascular‐Related NAC‐Domain 6 (VND6). VND6 is a master transcriptional regulator of secondary cell wall biosynthesis specifically in the xylem (Zhong, Lee, Zhou, McCarthy, & Ye, 2008). VND6 modulates secondary cell wall biosynthesis by activating the expression of several MYB transcription factor genes, secondary cell wall biosynthesis enzyme genes, and degradative enzyme genes involved in programmed cell death (Zhong et al., 2008). In roots from NAE 18:2‐ or NAE 18:3‐treated seedlings, transcripts that encode MYB transcription factors, previously shown to be modulated by VND6 (Zhong et al., 2008), also were less abundant. In addition, NAE 18:3 treatment led to a decrease in transcripts that encode MYB83 and MYB46 in roots. These two transcription factors also modulate secondary cell wall biosynthesis by activating additional transcription factors and secondary cell wall biosynthesis enzymes (Zhong & Ye, 2012). Collectively, these gene expression changes induced by NAE 18:2 or NAE 18:3 treatment in roots are consistent with an overall inhibition in secondary cell wall biosynthesis and reduction in vascular development, a structural change likely associated with reduced primary root elongation.

In addition to identifying genes that may initiate the downstream phenotypic changes, our detailed transcriptome analyses also identified potential targets of NAE signaling that may be directly involved in modulating growth (Figure 10a). NAE 18:3 treatment led to a decrease in transcripts that encode CESA7 or CESA4, specifically in roots. NAE 18:2 treatment also led to a decrease in transcripts that encode CESA7 in intact seedlings. These two cellulose syntheses are non‐redundantly required for secondary cell wall biosynthesis (Li, Bashline, Lei, & Gu, 2014). In addition, NAE 18:3 also led to a decrease in transcripts that encode CESA5, a cellulose synthase enzyme, that is redundant with CESA6 and involved in primary cell wall biosynthesis (Li et al., 2014), specifically in cotyledons. Exogenous treatment with NAE 18:2 and NAE 18:3 also led to a decrease in transcripts that encode non‐CESA genes involved in cellulose biosynthesis. NAE 18:2 and NAE 18:3 treatment led to a decrease in transcripts that encode the COBRA protein (COB1). COB1 is involved in the organization or orientation of cellulose microfibrils (Roudier et al., 2005). NAE 18:3 treatment also led to a decrease in transcripts that encode a COB1 paralog, COBRA‐like 4 (COBL4) or Chitinase‐like Protein 1 (CTL1). In addition to decreasing transcripts that encode proteins involved in cellulose biosynthesis, NAE 18:3 also significantly decreased the abundance of transcripts that encode STELLO1 (STL1). STELLO proteins affect the assembly of CESA complexes and the trafficking of these complexes from the Golgi apparatus to the plasma membrane (Zhang et al., 2016).

Exogenous treatment with NAE 18:2 and NAE 18:3 also led to decreases in transcripts encoding several enzymes involved in the metabolism of cell wall matrix polymers. For example, NAE 18:2 and NAE 18:3 led to decreases in transcripts that encode XTHs, XETs, and a XUT. These enzymes are involved in the integration of xyloglucan into the cell wall or the restructuring of xyloglucan within the cell wall (Becnel, Natarajan, Kipp, & Braam, 2006). Although there are 33 XTHs encoded by the *Arabidopsis* genome, members of this group of cell wall modifying enzymes have unique expression patterns with extensive overlap through every developmental stage (Becnel et al., 2006). NAE 18:2 and NAE 18:3 treatment led to lipid signal‐type and organ‐specific changes in transcripts that encode XTHs. In addition, transcripts that encode several enzymes involved in pectin metabolism were also significantly decreased as a result of NAE 18:2 and NAE 18:3 treatment.

The abundance of transcripts that encode structural components of cell walls was also significantly decreased as a result of NAE 18:2 and NAE 18:3 treatment. Both of these treatments led to a decrease in transcripts that encode cell wall structural proteins, including expansins and extensins. In addition, the abundance of transcripts that encode arabinogalactan proteins was also lower as a result of NAE 18:2 and NAE 18:3 treatment. A decrease in abundance of transcripts that encode transcriptional regulators of cell wall biosynthesis, cell wall enzymes, and structural constituents of cell walls suggested that NAE 18:2 and NAE 18:3 treatment inhibited growth as a result of a decrease in the abundance of cell wall proteins that are directly or indirectly involved in cell growth and expansion (Figures 10 and 12).

In conclusion, although our results provided insight into the lipid signal‐type and organ‐ specific transcriptional changes induced by NAE 18:2 and NAE 18:3, transcriptome changes alone do not provide a complete description of what is happening at the cellular and molecular level. The proteins encoded by these transcripts, the significantly enriched pathways, and the potential downstream molecular targets should be further investigated in order to determine how they are involved in NAE 18:2 and NAE 18:3 signaling pathways. In addition, results presented here showed that the transcripts modulated by NAE 18:2 and NAE 18:3 are involved in multiple stress response pathways. Understanding what environmental conditions result in an increase in the endogenous concentration of these NAEs will expand our understanding of the role that these lipophilic signals play in plant growth and development. The transcriptome data also reinforced the spatial aspects of this signaling pathway indicating organ‐specific responses to NAEs. In future studies, it will be important to explore the localization of NAE types themselves to provide further insight into the bifurcating nature of this pathway.

## CONFLICT OF INTEREST

The authors declare no conflict of interest.

## AUTHOR CONTRIBUTIONS

AEC and KDC designed the experiments; AEC performed the experiments and wrote the manuscript; CY assisted with experimental design and provided preliminary data and key insights into transcriptional changes induced by NAE18:3; DJB, XR, and RKA provided assistance with bioinformatics and statistical analyses; KDC supervised the project. All authors read and approved the manuscript.

## Supporting information

Supplementary Material

Supplementary Material

Supplementary Material

Supplementary Material

Supplementary Material

Supplementary Material

Supplementary Material

Supplementary Material

Supplementary Material

Supplementary Material

Supplementary Material

Supplementary Material

Supplementary Material

Supplementary Material

Supplementary Material

Supplementary Material
