# Supplementary figures and images for: Lipophilic signals lead to organ‐specific gene expression changes in Arabidopsis seedlings
Source: Plant Direct. 2020 Jul 15;4(7):e00242. doi: 10.1002/pld3.242 (PMC7403840; doi:10.1002/pld3.242)

Samples PCA

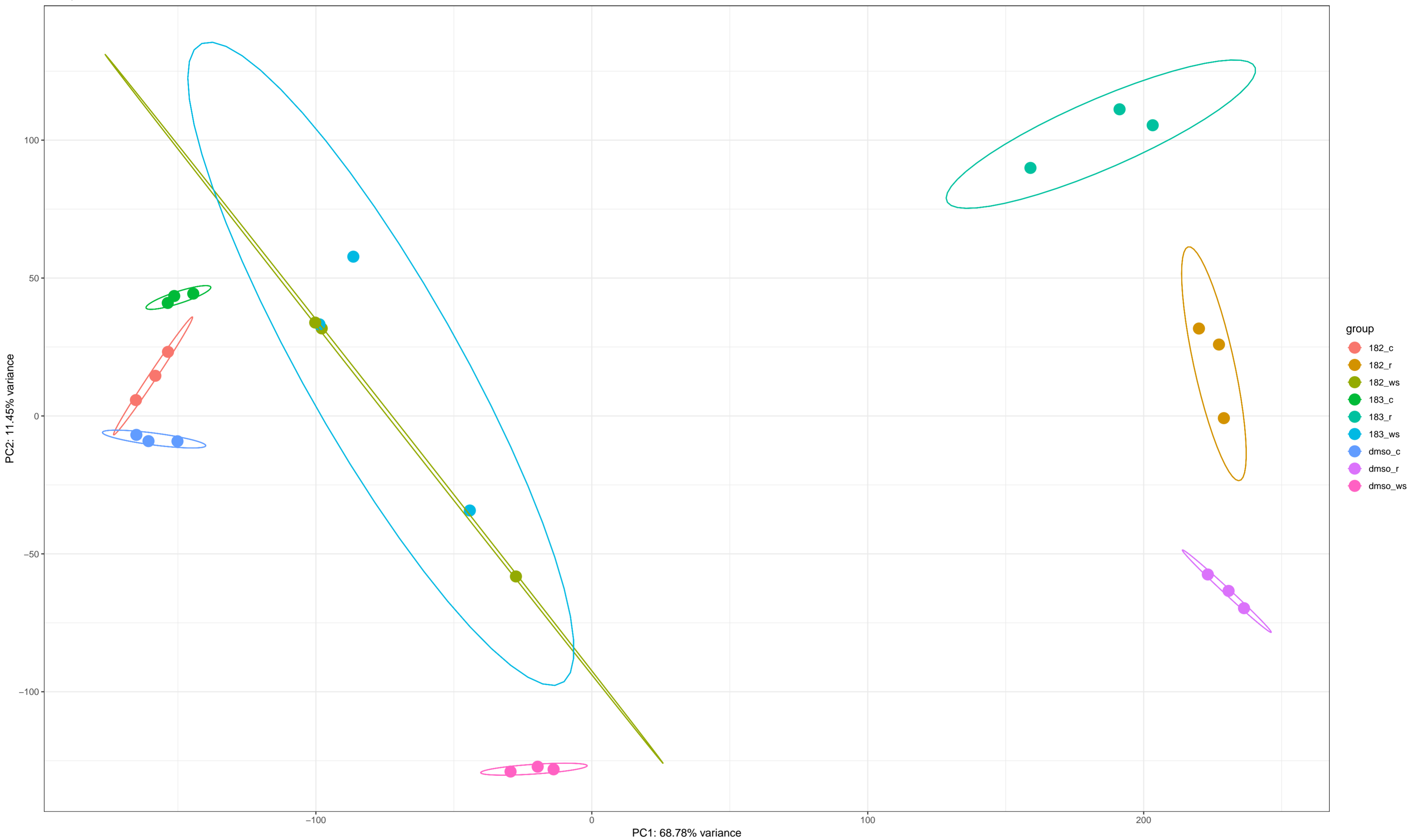

Supplement: Supplementary file 12 — Supplementary Material [file PLD3-4-e00242-s013.pdf]
